# *CD44* Gene rs8193 C Allele Is Significantly Enriched in Gastric Cancer Patients

**DOI:** 10.22074/cellj.2020.6389

**Published:** 2019-07-31

**Authors:** Roya Mokhtarian, Hossein Tabatabaeian, Pardis Saadatmand, Mansoureh Azadeh, Negar Balmeh, Bagher Yakhchali, Kamran Ghaedi

**Affiliations:** 1 Division of Cellular and Molecular Biology, Department of Biology, NourDanesh Institute of Higher Education, Meymeh, Iran; 2 Department of Biochemistry, Yong Loo Lin School of Medicine, National University of Singapore, Singapore; 3 Department of Biology, Faculty of Sciences, University of Isfahan, Isfahan, Iran; 4 ZistFanavari Novin Biotechnology Institute, Isfahan, Iran; 5 Institute of Industrial and Environmental Biotechnology, National Institute of Genetic Engineering and Biotechnology Institute, Tehran, Iran; 6 Department of Cellular Biotechnology, Cell Science Research Center, Royan Institute for Biotechnology, ACECR, Isfahan, Iran

**Keywords:** *CD44*, Gastric Cancer, miR-570

## Abstract

**Objective:**

Gastric cancer is a multifactorial disease. In addition to environmental factors, many genes are involved in this malignancy. One of the genes associated with gastric cancer is *CD44* gene and its polymorphisms. *CD44* gene plays role in regulating cell survival, growth and mobility. The single nucleotide polymorphism (SNP) rs8193, located in the *CD44* gene, has not been studied in gastric cancer patients of the Iranian population. The present study aims to study this polymorphism in 86 gastric cancer patients and 96 healthy individuals.

**Materials and Methods:**

In this cross-sectional case-control study, rs8193 polymorphism was genotyped by allele specific primer polymerase chain reaction (ASP-PCR) technique. The obtained data were statistically analyzed. To find the potential mechanism of action, rs8193 was bioinformatically investigated.

**Results:**

rs8193 C allele played a risk factor role for gastric cancer. Patients carrying this allele were more susceptible to have gastric cancer, with lymph node spread. On the other hand, rs8193 T allele, a protective factor, was associated with a higher chance of accumulation in the lower stages of cancer. C allele might impose its effect via destabilizing CD44 and miR-570 interaction.

**Conclusion:**

rs8193 is statistically associated with the risk of malignancy, lymph node spread and stage of gastric cancer in Iranian population.

## Introduction

Gastric cancer is one of the most prevalent and leading causes of cancer death (1). Several factors such as race, ethnicity, sex, age, genetic and environmental factors are associated with this malignancy. The International Agency for Research on Cancer reported *Helicobacter pylori (H. pylori)* infection as the potential risk factor for gastric adenocarcinoma and described it as a group 1 carcinogen (2). The key genes involved in the development of cancer include the oncogenes and tumor suppressor genes, contributing to DNA repair and apoptosis mechanisms, among which *K-Ras, Myc* and *CD44* are the most important genes that have been proven to be associated with gastrointestinal cancers (3).

From the cytogenetic point of view, *CD44* gene is located on chromosome 11p13 (4). This gene encodes various protein isoforms generated by alternative processing and post-translation modifications. CD44 and its processed isoforms are central mediators of cellular behaviors, such as cell survival, growth and mobility. Interest in the study of CD44 was boosted when CD44v6 was confirmed to induce complete metastasis in non-metastatic cancer of the rat pancreatic cells. It has increasingly been shown that CD44v6 is expressed from gastric precancerous lesions to advanced carcinoma, while role of CD44 in tumorigenesis still remains controversial (5). 

Almost all cell-signaling pathways are regulated by microRNAs (miRNAs) and consistently physiological phenotypes of stomach cells are regulated by these small non-coding RNAs. Some of these miRNAs target the oncogenes, known as tumor suppressor miRNAs (tsmiRs), while the others modulate expression of tumor suppressor genes and known as oncomiRs (6-10). In addition, evidences demonstrate the regulatory effect of miRNAs during carcinogenesis, through modulating cell proliferation, migration, invasion and anti-apoptotic properties (11-13).

*CD44* is one of the genes tightly correlated with gastric cancer (14). The association of miRNA-related single nucleotide polymorphism (SNP) rs8193, located within 3´UTR of *CD44* gene, has not been studied in gastric cancer of Iranian population. Therefore, in this study, we aimed to investigate frequency of different rs8193 alleles in Iranian population. We further conducted in silico study in order to have a predicted vision on a mechanism of action, whereby different rs8193 alleles can alter the carcinogenic impact of *CD44* gene.

## Materials and Methods

### Compliance with ethical standards 

All procedures performed in this study including human participants and ethical considerations were on the basis of the Ministry of Health and Medical Education of Iran and the 1964 Helsinki declaration. The study protocol was approved by the Nourdanesh Institute of Higher Education in Meymeh, Iran. Informed consent was obtained from all participants involved in this study. 

### Study subjects

**Fig.1:**
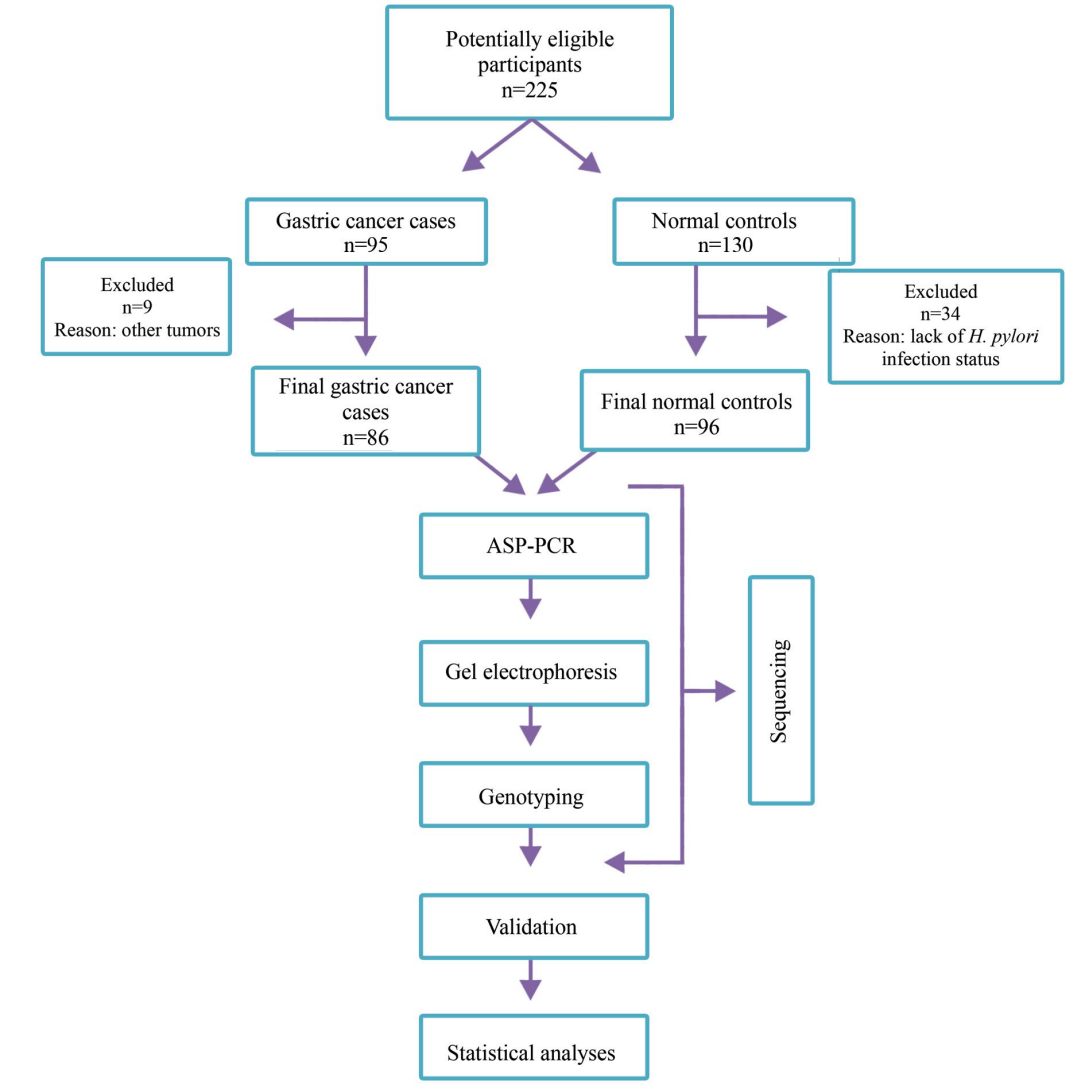
STARD diagram reporting flow of participants. ASP-PCR; Allele-specific primer polymerase chain reaction.

In this cross-sectional case-control study, 5 ml of peripheral blood samples were taken from 86 gastric cancer patients and 96 healthy controls in Seyed-al- Shohada Hospital, Isfahan, Iran. All participants were selected randomly and analyzed in advance (Fig .1). To prevent clotting, the blood samples were transferred into tubes containing 1 ml EDTA at a concentration of 0.5 M, and then stored at -20°C until testing. The presence of *H. pylori* infection was qualitatively evaluated by an expert pathologist. 

### DNA extraction and primer designing

In this study, the PrimePrep Genomic DNA Isolation Kit from Blood (GeNetBio, Korea) was used for DNA extraction according to the manufacturer’s instructions. In addition, allele-specific primer polymerase chain reaction (ASP-PCR) method was applied to examine the SNP genotypes. Three primers, including wild-type forward, SNP forward and common reverse primer, were designed in accordance with the WASP site at http://bioinfo.biotec. or.th/WASP (Table 1) and they were ordered from Bioneer Company (Korea). 

**Table 1:** Primer sequences utilized for ASP-PCR


Primer	Sequence (5ˊ-3ˊ)

Wild-type forward	CCTAATCCCTGGGCACTGC
SNP forward	CATAGCCTAATCCCTGGGCATTAT
Common reverse	ATACATTGTAGGGACCCAGACAGTG


ASP-PCR; Allele-specific primer polymerase chain reaction and SNP; Single nucleotide polymorphism.

The primer-specific binding sites were confirmed by BLAST assay. The primers were designed to align 3’ terminal of both wild-type forward and SNP forward primers with the SNP site. A mismatch nucleotide was also designed at -2 and -4 nucleotide regions in order to minimize possibility of non-specific annealing, as described by Assad Samani et al. (15). 

### Genotyping by allele-specific primer polymerase chain reaction

ASP-PCR was performed in a final volume of 25 µl, as previously described (16, 17), including 4 µl DNA template, 2.5 µl of 10X PCR buffer (Bioron, Germany), 0.75 µl MgCl2 (50 mM), 1 µl dNTP mix (10 mM, both from Bioron, Germany), 1 µl wild-type forward primer (10 µM), 1 µl SNP forward primer (10 µM), 1 µl reverse primer (10 µM), 0.25 µl Taq DNA polymerase (5 U/µl, Bioron, Germany), and 14.5 µl distilled H_2_O.

Gradient temperature and MgCl_2_ was used to optimize the ASP-PCR conditions. The best-optimized conditions are as follows: hot start 95°C for 5 minutes, 35 cycles including denaturation at 94°C for 20 seconds, annealing at 58.5°C for 50 seconds, extension at 72°C for 50 seconds, and the final extension at 72°C for 10 minutes. PCR products were analyzed by 2% agarose gel electrophoresis and RedSafe Nucleic Acid Staining Solution (iNTRON, Hong Kong), in order to determine the genotypes. Dissimilar to tetra- primer ARMS-PCR, as a multiplex-based method (18), reactions were performed in two separate vials in ASP-PCR technique. Some random samples from both groups were sequenced and the outcomes confirmed the accuracy of genotyping performed by ASP-PCR technique. 

### Statistical analysis

The univariate (Chi-square test and Fisher’s exact test) and multivariate (Multivariate logistic regression) analyses were performed using SPSS software (version 19, SPSS Inc., USA). For all tests, P<0.05 was considered statistically significant.

### Data sources

Interaction analysis between miRNA and SNP rs8193, located in *CD44* 3’UTR of respective mRNA, was performed by miRNASNP database V2.0 to identify the potential miRNAs with capability of targeting 3’UTR of *CD44* transcripts. This database was also used to predict the effect of different rs8193 alleles on the binding affinity of miRNA570 to *CD44* transcript, as well as modulation in gibbs free energy (ΔG) of binding reaction (19, 20). 

### Single nucleotide polymorphism data

Critical information on rs8193, such as the minor allele frequency (MAF) as well as upstream and downstream sequences of this SNP, was obtained from the NCBI database (http://www.ncbi.nlm.nih.gov).

### Signaling system enrichment analysis 

A possible target of rs8193 associated with miRNA570 was obtained using miRWalk V2.0 database (21). Finally, the database for annotation, visualization and integrated discovery (DAVID) V6.7 was used for cell signaling enrichment analyses (22, 23).

## Results

### Genotyping the rs8193 position in control and gastric cancer samples 

**Fig.2:**
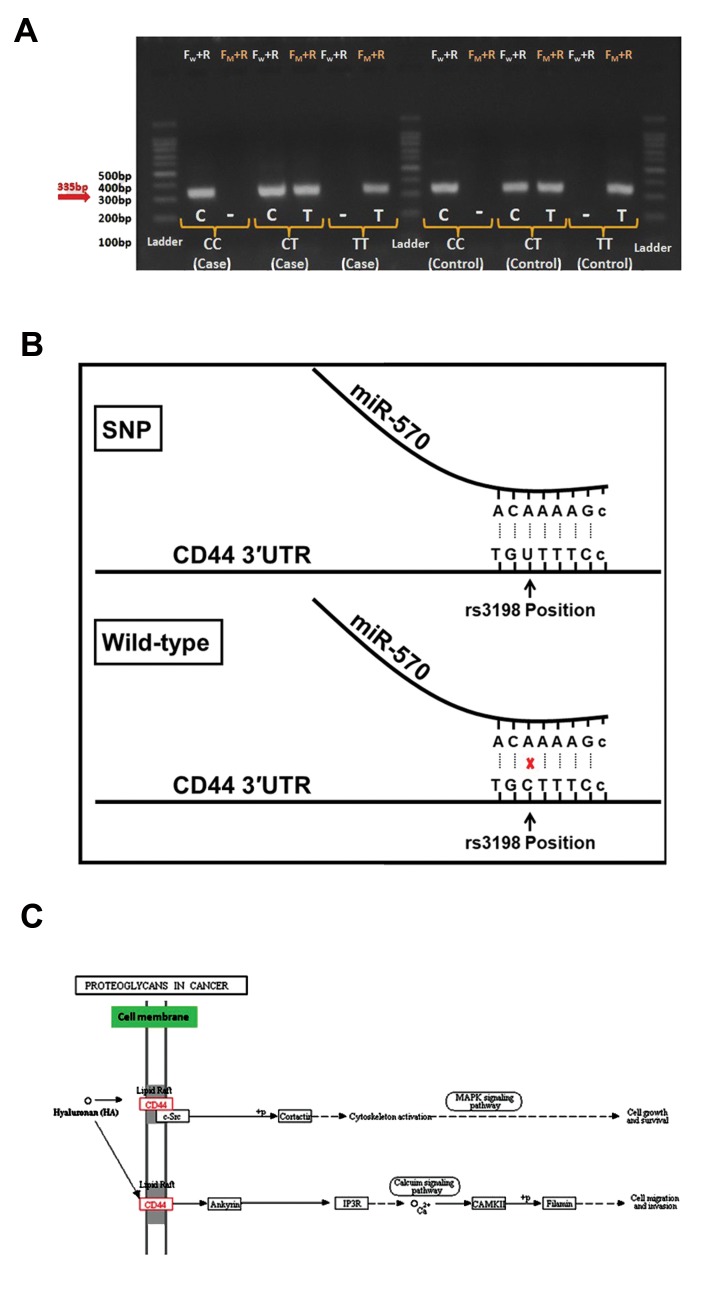
Molecular characteristics of rs3198 position and genotyping of the samples. **A.** Optimized ASP-PCR followed by gel electrophoresis, **B.** The schematic view of allele T and C effect on the interaction between miR570 and *CD44* mRNA at rs3198 position, and **C.** Enrichment analysis of miR-570 and its importance in targeting *CD44*.

Due to almost similarity of the fragment sizes, two separate ASP-PCRs were performed: one with wild- type forward and reverse primers and the other with SNP forward and reverse primers. Using agarose gel electrophoresis, two bands of 335 and 340 bp in heterozygous (CT) individuals, one band of 335 bpin homozygous dominant (CC) and one band of 340bp in homozygous recessive (TT) was observed. Control samples with two bands of 335 and 340 bp, relating respectively to C and T alleles, demonstratedCT genotype. Control samples with only 335 bp bandrepresented CC genotype, and control samples withonly 340 bp revealed TT genotype (Fig .2A). Somesamples were sequenced to validate the efficacy of theASP-PCR. The outcomes of sequencing were totally consistent with the ASP-PCR findings, confirming the efficacy of the ASP-PCR method.

### rs8193 C allele associates with the risk of gastric cancer 

To study association of different rs8193 genotypes with risk of the gastric cancer, we studied the samples in two ways. First, we considered allele T as the risk variant. Therefore, the samples were analyzed as CC compared to CT+TT (recessive model). The statistical outcomes revealed no significant association (Pearson chi-square test, P=0.122). However, categorizing CC+CT samples, as dominant model compared to TT, showed a significant association (Pearson chi- square test, P<0.001). This outcome indicated that harboring allele C has increased the risk of gastric cancer with odds ratio (OR) of 3.429 [95% confidence interval (CI): 1.768-6.647]. Univariate association study between the patients carrying C allele and the incidence of gastric cancer is shown in Table 2. 

To evaluate the significance of rs8193 C allele risk in the studied gastric cancer patients, we incorporated confounder factors into the regression model. These factors consist of blood groups, smoking status and *H. pylori* infection (Table 3). Based on the univariate analysis (Table 2), *H. pylori* infection and harboring C allele at the rs8193 position were both associated with gastric cancer outcome. Although statistical analysis showed that *H. pylori* infection had higher significance to associate with the gastric cancer outcome, benefiting from the multivariate logistic regression. Study the effect of rs8193 in the context of other confounders revealed that rs8193 C allele was still associated with the increased risk of gastric cancer with OR: 2.888 (95% CI: 1.430-5.835, P=0.003). Wald value for *H. pylori* was 16.707; while it was 8.742 for carrying C allele, showing the higher significance of *H. pylori* infection to contribute to the gastric cancer outcome. As both *H. pylori* infection and rs8193 C allele were significantly important to have the gastric cancer outcome, we performed a chi-square test in order to know if rs8193 is associated with risk of* H. pylori* infection. Among 24 TT samples, 12 were *H. pylori* positive and 12 samples were negative. Among 144 C allele carriers, 119 cases did not show *H. pylori* infection and 91 samples showed *H. pylori* infection. Chi-square test revealed that there is no association between *H. pylori* infection and rs8193 C allele; therefore, rs8193 C allele does not correlate with the risk of *H. pylori*-mediated gastric cancer (P=0.534). Taken together, these data strongly suggest that the C allele carriers at rs8193 position are genetically predisposed to gastric cancer. 

Based on the available clinicopathological characteristics of the gastric cancer patients, we investigated if having C allele at rs8193 position would associate with distal metastasis, lymph node spread and stage of gastric cancer. Among different conditions, statistical analyses demonstrated that the patients who carried C allele associated with higher chance of having regional lymph node spread with OR: 4.896 (95% CI: 1.985-12.076, P<0.001, Table 4). Moreover, these patients were less likely to have stage I gastric cancer, (OR: 0.241, 95% CI: 0.084-0.688, P=0.011), as C allele-carriers are more accumulated in the groups with higher stages. Clinically, these findings support the prognostic importance of rs8193 C allele in gastric cancer. 

**Table 2:** Univariate comparison of the controls and cases


Variable		Cancer	Controls	OR (95% CI)	P value^*^
		n=168	n=66		
		n (%)	n (%)		

Smoking				-	0.748
	No	75 (44.64)	31 (46.97)		
	Yes	93 (55.36)	35 (53.03)		
*H. pylori* infection				4.704 (2.388-9.268)	< 0.001
	No	78 (46.43)	53 (80.30)		
	Yes	90 (53.57)	13 (19.70)		
Blood group A				-	0.926
	No	108 (64.29)	42 (63.64)		
	Yes	60 (35.71)	24 (36.36)		
Carrying C allele at rs8193 position				3.429 (1.768-6.647)	< 0.001
	No	24 (14.29)	24 (36.36)		
	Yes	144 (85.71)	42 (63.64)		


*; Chi-square test, OR; Odds ratio, and CI; Confidence interval.

**Table 3:** Multivariate logistic regression comparison of the controls and cases


Variable		Cancer n=168	Controls n=66	OR (95% CI)	P value^*^

Smoking				-	0.786
	No	75	31		
	Yes	93	35		
*H. pylori* infection				4.223 (2.116-8.425)	< 0.001
	No	78	53		
	Yes	90	13		
Blood group A			-	0.957
	No	108	42		
	Yes	60	24		
Carrying C allele at rs8193 position				2.888 (1.430-5.835)	0.003
	No	24	24		
	Yes	144	42		


*; Multivariate logistic regression. Smoking (No), H. pylori infection (No), blood group A (No) and carrying C allele at rs8193 position (No) were considered as references of cancer outcom, OR; Odds ratio, and CI; Confidence interval.

**Table 4:** Association of rs8193 C allele harboring patients with gastric cancer characteristics


Characteristic	rs8193 genotype		OR (95% CI)	P value
	TT n=24	CC+CT n=144		
n (%)	n (%)

Distal metastasis				0.899^*^
No	13 (54.17)	80 (55.56)		
Yes	11 (45.83)	64 (44.44)		
Regional lymph node spread			4.896 (1.985-12.076)	<0.001^*^
No	13 (54.17)	28 (19.44)		
Yes	11 (45.83)	116 (80.56)		
Stage			0.241 (0.084-0.688)	0.011^**^
I	7 (29.17)	13 (9.03)		
II, III and IV	17 (70.83)	131 (90.97)		
II	2 (8.33)	30 (20.83)	-	0.254^**^
I, III and IV	22 (91.67)	114 (79.19)		
III	6 (25)	48 (33.33)	-	0.487^**^
I, II and IV	18 (75)	96 (66.67)		
IV	9 (37.50)	53 (36.81)	-	0.998^**^
I, II and III	15 (62.50)	91 (63.19)		


*; Chi-square test, **; Fisher’s exact test, OR; Odds ratio, and CI; Confidence interval.

### Potential role of rs8193 in modulating interaction of *CD44* mRNA with miRNAs

As rs8193 is located in the 3ˊUTR of *CD44* gene, we postulated that this SNP may impose its effect through altering interaction of *CD44* mRNA with miRNAs. Using miRNASNP online database, exploring potential of the different rs8193 alleles demonstrated disrupting role of C allele, with regards to the interaction between *CD44* mRNA and *miR-570*. Indeed, C allele, which is shown to be a risk factor in this study, could result in higher expression of *CD44*, due to the lower binding affinity of *miR-570* to it. On the other hand, rs8193 T allele, as a protective factor, can strengthen the interaction and therefore reduce the *CD44* expression level (Fig .2B). This finding is supported by the changes in ΔG values of *miR- 570:CD44* interaction. This interaction could be stable (ΔG=-13.70) when T is positioned, while it is unstable when C is replaced (ΔG=0).

Study the targetome of *miR-570* in miRWalk database further showed *CD44* as one of the high-score predicted genes, with the score 6 out of 7 integrated algorithms used (Table S1) (See Supplementary Online Iiformation at www.celljournal.org). Moreover, enrichment analysis of miR-570 targetome in DAVID tool suggested *CD44* as a putative target in gastric carcinogenesis. As shown in Figure 2C, activity of the CD44 along with proteoglycan hyaluronan (HA) in MAPK signaling pathway is associated with growth and survival of tumor cells [P=1.5×10^-6^, false discovery rate (FDR) correction=0.0002]. Moreover, this interaction can result in cellular invasion and migration via calcium signaling pathway (P=5.5×10^-7^, FDR correction=0.0001). Albeit the in silico studies are required to be validated by the biochemical assays, these data are quite compatible with the outcomes of our study, showing that C allele is the risk factor. Rs8193 C allele, indeed, can attenuate the interaction between *miR-570* and *CD44* mRNA, which in turn, enhances the expression and oncogenic effect of CD44 in gastric cells. 

## Discussion

Late diagnosis, complexity of treatment and prevention are the main concerns of gastric cancer. These issues are tightly related to the multifactorial nature of this disease. Therefore, early detection of gastric cancer is highly necessary in order to control it. Along with the environmental parameters like *H. pylori* infection, genetic factors including gene expression profile and cancer biomarkers, such as SNPs, have a crucial role to better predict early diagnosis of gastric cancer (17). 

In this study, we genotyped the rs8193 position in control and gastric cancer samples. Although the main cause of gastric cancer in the studied population was *H. pylori* infection, rs8193 C allele was shown to be associated with higher risk of gastric cancer, in both univariate and multivariate logistic regression models. Clinically, rs8193 C allele also associated with the enhanced risk of regional lymph node spread and lower chance of categorization in the gastric cancer stage I. In order to have an improved vision of rs8193 potential, it is highly recommended to study this SNP in a larger population with more patients’ demographics, such as age, sex, alcohol consumption status, occupation, etc. 

CD44, as a type 1 of single-pass transmembrane protein, is an important adhesive molecule for the extracellular matrix. It acts as a cell surface receptor of hyaluronic acid, and interferes with various biological processes such as cell adhesion, cell migration and cancer metastasis. In addition, *CD44* gene may increase the risk of tumor recurrence in a variety of cancers (24).

In order to find the potential role of rs8193 C allele in increasing chance of gastric cancer, *in silico* studies were recruited. Regarding that rs8193 is located within *CD44* 3’UTR, alteration in miRNA binding affinity could be the main mechanism of action for different rs8193 alleles. Based on the investigations, rs8193 C allele can attenuate the interaction between *miR-570* and *CD44* mRNA, which may result in the higher expression of CD44 oncogene. Interestingly, Mumbrekar et al. (25) have shown that rs8193 can alter the expression of CD44, based on the HapMap database results. Moreover, functional SNP dataset (F-SN) indicated that rs8193 might change the affinity of transcription factors to the CD44 promoter, due to a distant conformational effect. Taken together, they have also concluded that rs8193 is important for regulating expression of CD44. These data strongly confirm the in silico outcomes obtained from our study. 

Enrichment analysis of *miR-570* targetome further revealed that this small non-coding RNA can target *CD44*, imposing its oncogenic role through MAPK and calcium signaling pathways. Therefore, rs8193 C allele, associated with higher risk of gastric cancer, might destabilize *CD44* and *miR-570* interaction. This, in turn, results in higher expression and oncogenic impact of CD44. These mechanistic postulations are highly needed to be validated by luciferase reporter assay, Quantitative real time polymerase chain reaction (qRT-PCR) and western blot methods, which were out of the amenities of this study.

Albeit this proposed model can describe the potential mechanism of action by which rs8193 allele C can impose its oncogenic effect in gastric cancer, *in vitro CD44* 3´UTR luciferase assay with two alleles T and C and in the presence and absence of miR-570 mimic, is strongly recommended in order to validate the ability of *miR-570* to differentially target *CD44* mRNA. Furthermore, CD44 protein expression could be evaluated by western blot, immunohistochemistry (IHC) or ELISA in the samples with T and C alleles to understand if the CD44 expression is truly altered in samples with different genotypes, in the presence of *miR-570* mimics or related inhibitors. 

The polymorphisms affecting interaction affinity of *miR-570* with its target genes have been reported in gastric cancer. Previous studies have shown that polymorphisms in the binding site of *miR-570* to the *B7-H1* and *CD274* genes associates with the risk of gastric cancer and it might involve in human cancers (26, 27). Moreover, another study found that *miR-570-3p* is one of the diagnostic biomarkers for asthma and it is a potential pro- inflammatory miRNA. This miRNA up-regulates various types of cytokines as well as chemokines (CCL4, CCL5, TNFα and IL-6) and increases their induction by TNFα. It also has an inhibitory effect to suppress up-regulation of other cytokines (CCL2 and IL-8) by TNFα (28). 

Evidences show that the other *CD44* SNPs could also contribute to cancer. Studies showed that the polymorphisms in *CD44* play a substantial role in the development of breast (29) and bladder cancers (30) in the northern Indian population and they may be important as a molecular prognostic markers. Moreover, CD44 haplotypes have been shown to significantly associate with the increased incidence of gastric cancer in Chinese patients (24). Furthermore, there is a significant relationship between the *CD44* rs187115 and liver cancer (31).

Another investigation demonstrated that various SNPs of *CD44* gene, including rs8193, have a significant association with gastric cancer in the Chinese population. According to this study, there was a significant association between rs8193 TT genotype (reported as a protective genotype in our study) and higher chance for lower tumor size or lower serosal invasion (32). 

Collecting findings of the current and other studies have shown the importance of rs8193 in cancer, especially gastric malignancy. Further studies in a larger scale with taking the advantage of validation of the proposed *miR-570*-mediated mechanistic effect of rs8193 on the expression of *CD44* can more clarify the significance of different rs8193 alleles in cancer. 

## Conclusion

An SNP located within 3´UTR of *CD44*, rs8193, statistically associate with the risk of lymph node spread and stage of gastric cancer in Iranian population. In this study, rs8193 C allele has been introduced as a risk allele. This allele associates with higher risk of gastric cancer. Distribution of C allele is also enriched in the patients with regional lymph node metastasis. On the other hand, T allele plays role, as a protective allele, and it is statistically enriched in the gastric cancer patients with lower stage. 

## Supplementary PDF



## References

[1] Garay J, Piazuelo MB, Majumdar S, Li L, Trillo-Tinoco J, Del Valle L (2016). The homing receptor CD44 is involved in the progression of precancerous gastric lesions in patients infected with Helicobacter pylori and in development of mucous metaplasia in mice. Cancer Lett.

[2] Baroudi O, Benammar-Elgaaied A (2016). Involvement of genetic factors and lifestyle on the occurrence of colorectal and gastric cancer. Crit Rev Oncol Hematol.

[3] Croce CM (2008). Oncogenes and Cancer. N Engl J Med.

[4] Cooper DL, Dougherty G, Harn HJ, Jackson S, Baptist EW, Byers J (1992). The complex CD44 transcriptional unit: alternative splicing of three internal exons generates the epithelial form of CD44. Biochem Biophys Res Commun.

[5] Branco da Cunha C, Klumpers DD, Koshy ST, Weaver JC, Chaudhuri O, Seruca R (2016). CD44 alternative splicing in gastric cancer cells is regulated by culture dimensionality and matrix stiffness. Biomaterials.

[6] Dehghan Z, Sadeghi S, Tabatabaeian H, Ghaedi K, Azadeh M, Fazilati M (2017). ESR1 single nucleotide polymorphism rs1062577 (c.* 3804T> A) alters the susceptibility of breast cancer risk in Iranian population. Gene.

[7] Zabihi N, Sadeghi S, Tabatabaeian H, Ghaedi K, Azadeh M, Fazilati M (2017). The association between rs1972820 and the risk of breast cancer in Isfahan population. J Cancer Res Ther.

[8] Salimi Z, Sadeghi S, Tabatabaeian H, Ghaedi K, Fazilati M (2016). rs11895168 C allele and the increased risk of breast cancer in Isfahan population. Breast.

[9] Rouigari M, Dehbashi M, Tabatabaeian H, Ghaedi K, Mohammadynejad P, Azadeh M (2018). Evaluation of the expression level and hormone receptor association of miR-126 in breast cancer.Indian J Clin Biochem.

[10] Mansouri Bidkani M, Tabatabaeian H, Parsafar S, Ghanei N, Fazilati M, Ghaedi K (2018). ErbB4 receptor polymorphism 2368A> C and risk of breast cancer. Breast.

[11] Noormohammad M, Sadeghi S, Tabatabaeian H, Ghaedi K, Talebi A, Azadeh M (2016). Upregulation of miR-222 in both Helicobacter pylori-infected and noninfected gastric cancer patients. J Genet.

[12] Adami B, Tabatabaeian H, Ghaedi K, Talebi A, Azadeh M, Dehdashtian E (2018). miR-146a is deregulated in gastric cancer. J Cancer Res Ther.

[13] Garzon R, Calin GA, Croce CM (2009). MicroRNAs in cancer. Annu Rev Med.

[14] Wakamatsu Y, Sakamoto N, Oo HZ, Naito Y, Uraoka N, Anami K (2012). Expression of cancer stem cell markers ALDH1, CD44 and CD133 in primary tumor and lymph node metastasis of gastric cancer. Pathol Int.

[15] Assad Samani L, Javadirad SM, Parsafar S, Tabatabaeian H, Ghaedi K, Azadeh M (2018). TP53 rs1625895 is related to breast cancer incidence and early death in Iranian population.Indian J Clin Biochem.

[16] Moradi B, Tabatabaeian H, Sadeghi S, Azadeh M, Ghaedi K (2016). HER4 rs1595065 3’UTR variant is a possible risk factor for HER2 Positivity among breast cancer patients. Thrita.

[17] Nabatchian F, Rahimi Naiini M, Moradi A, Tabatabaeian H, Hoghoughi N, Azadeh M (2018). miR-581-related single nucleotide polymorphism, rs2641726, located in MUC4 gene, is associated with gastric cancer incidence.Indian J Clin Biochem.

[18] Honardoost MA, Tabatabaeian H, Akbari M, Salehi M (2014). Investigation of sensitivity, specificity and accuracy of Tetra primer ARMS PCR method in comparison with conventional ARMS PCR, based on sequencing technique outcomes in IVS-II-I genotyping of beta thalassemia patients. Gene.

[19] Gong J, Tong Y, Zhang HM, Wang K, Hu T, Shan G (2012). Genomewide identification of SNPs in microRNA genes and the SNP effects on microRNA target binding and biogenesis. Hum Mutat.

[20] Tabatabian M, Mesrian Tanha H, Tabatabaeian H, Sadeghi S, Ghaedi K, Mohamadynejad P (2018). ErbB4 3′-UTR variant (c.* 3622A> G) is associated with ER/PR negativity and advanced breast cancer. Indian J Clin Biochem.

[21] Dweep H, Gretz N (2015). miRWalk2.0: a comprehensive atlas of microRNA- target interactions. Nat Methods.

[22] Huang da W, Sherman BT, Lempicki RA (2009). Bioinformatics enrichment tools: paths toward the comprehensive functional analysis of large gene lists. Nucleic Acids Res.

[23] Noormohammad M, Khatami M, Tabatabaeian H, Ghaedi K, Talebi A, Heidari MM (2014). In-silico investigation of Mir-222 in H.Pylori-associated gastric cancer. Iranian Journal of Public Health.

[24] Verma A, Kapoor R, Mittal RD (2017). Cluster of differentiation 44 (CD44) gene variants: a putative cancer stem cell marker in risk prediction of bladder cancer in north Indian population. Indian J Clin Biochem.

[25] Mumbrekar KD, Bola Sadashiva SR, Kabekkodu SP, Fernandes DJ, Vadhiraja BM, Suga T (2017). Genetic variants in CD44 and MAT1A confer susceptibility to acute skin reaction in breast cancer patients undergoing radiation therapy. Int J Radiat Oncol Biol Phys.

[26] Wang W, Li F, Mao Y, Zhou H, Sun J, Li R (2013). A miR-570 binding site polymorphism in the B7-H1 gene is associated with the risk of gastric adenocarcinoma. Hum Genet.

[27] Wang W, Sun J, Li F, Li R, Gu Y, Liu C (2012). A frequent somatic mutation in CD274 3′‐UTR leads to protein over‐expression in gastric cancer by disrupting miR‐570 binding. Hum Mutat.

[28] Roff AN, Craig TJ, August A, Stellato C, Ishmael FT (2014). MicroRNA- 570-3p regulates HuR and cytokine expression in airway epithelial cells. Am J Clin Exp Immunol.

[29] Tulsyan S, Agarwal G, Lal P, Agrawal S, Mittal RD, Mittal B (2013). CD44 gene polymorphisms in breast cancer risk and prognosis: a study in North Indian population. PLoS One.

[30] Sharma KL, Yadav A, Gupta A, Tulsayan S, Kumar V, Misra S (2014). Association of genetic variants of cancer stem cell gene CD44 haplotypes with gallbladder cancer susceptibility in North Indian population. Tumour Biol.

[31] Chou YE, Hsieh MJ, Chiou HL, Lee HL, Yang SF, Chen TY (2014). CD44 gene polymorphisms on hepatocellular carcinoma susceptibility and clinicopathologic features. Biomed Res Int.

[32] Qiu Y, Hu Y, Zhang Z-Y, Ye L, Xu F-H, Schneider ME (2014). Genetic association of osteopontin (OPN) and its receptor CD44 genes with susceptibility to Chinese gastric cancer patients. J Cancer Res Clin Oncol.

