# Supplementary material for: CD44 Gene rs8193 C Allele Is Significantly Enriched in Gastric Cancer Patients
Source: Cell J. 2019 Jul 31;21(4):451–8. doi: 10.22074/cellj.2020.6389 (PMC6722445; doi:10.22074/cellj.2020.6389)
Supplement: Supplementary file 1 [file Cell-J-21-451-s01.pdf]

CELL JOURNAL (Yakhteh)

www.celljournal.org

Supplementary Information for

CD44 Gene rs8193 C Allele Is Significantly Enriched in Gastric Cancer Patients

Roya Mokhtarian, M.Sc.[1], Hossein Tabatabaeian, Ph.D.[2, 3], Pardis Saadatmand, M.Sc.[4], Mansoureh Azadeh, M.Sc.[4], Negar Balmeh, M.Sc.[1], Bagher Yakhchali, Ph.D.[5], Kamran Ghaedi, Ph.D.[3, 6*]

1. Division of Cellular and Molecular Biology, Department of Biology, NourDanesh Institute of Higher Education, Meymeh, Iran
2. Department of Biochemistry, Yong Loo Lin School of Medicine, National University of Singapore, City????, Singapore
3. Department of Cellular Biotechnology, Cell Science Research Center, Royan Institute for Biotechnology, ACECR, Isfahan, Iran
4. ZistFanavari Novin Biotechnology Institute, Isfahan, Iran
5. Institute of Industrial and Environmental Biotechnology, National Institute of Genetic Engineering and Biotechnology Institute, Tehran, Iran
6. Department of Biology, Faculty of Sciences, University of Isfahan, Isfahan, Iran

**Corresponding Address: P.O.Box: 816513-1378, Department of Cellular Biotechnology, Cell Science Research Center, Royan Institute for Biotechnology, ACECR, Isfahan, Iran*
Email: kamranghaedi@royaninstitute.org

Table S1: miR-570 targetome

miRNA	Gene	miRWalk	Microt4	miRanda	miRMap	RNA22	RNAhybrid	Targetscan	SUM
miR-570-3p	*ACVR2A*	1	1	1	1	1	1	1	7
miR-570-3p	*ADCYAP1R1*	1	1	1	1	1	1	1	7
miR-570-3p	*ZFHX3*	1	1	1	1	1	1	1	7
miR-570-3p	*ATRX*	1	1	1	1	1	1	1	7
miR-570-3p	*ATRX*	1	1	1	1	1	1	1	7
miR-570-3p	*CAMK2A*	1	1	1	1	1	1	1	7
miR-570-3p	*CDK6*	1	1	1	1	1	1	1	7
miR-570-3p	*CEBPG*	1	1	1	1	1	1	1	7
miR-570-3p	*CPM*	1	1	1	1	1	1	1	7
miR-570-3p	*DLG2*	1	1	1	1	1	1	1	7
miR-570-3p	*EDNRB*	1	1	1	1	1	1	1	7
miR-570-3p	*ERCC4*	1	1	1	1	1	1	1	7
miR-570-3p	*FSHB*	1	1	1	1	1	1	1	7
miR-570-3p	*GCNT2*	1	1	1	1	1	1	1	7
miR-570-3p	*NR3C1*	1	1	1	1	1	1	1	7
miR-570-3p	*GSK3B*	1	1	1	1	1	1	1	7
miR-570-3p	*LIPA*	1	1	1	1	1	1	1	7
miR-570-3p	*LRP6*	1	1	1	1	1	1	1	7
miR-570-3p	*MARCKS*	1	1	1	1	1	1	1	7
miR-570-3p	*MDM4*	1	1	1	1	1	1	1	7
miR-570-3p	*MEF2C*	1	1	1	1	1	1	1	7
miR-570-3p	*MEF2D*	1	1	1	1	1	1	1	7
miR-570-3p	*KMT2A*	1	1	1	1	1	1	1	7
miR-570-3p	*MTM1*	1	1	1	1	1	1	1	7
miR-570-3p	*MTM1*	1	1	1	1	1	1	1	7
miR-570-3p	*NFATC2*	1	1	1	1	1	1	1	7
miR-570-3p	*NMT1*	1	1	1	1	1	1	1	7
miR-570-3p	*PBX1*	1	1	1	1	1	1	1	7
miR-570-3p	*PLXNA2*	1	1	1	1	1	1	1	7
miR-570-3p	*PMP2*	1	1	1	1	1	1	1	7
miR-570-3p	*KDM5A*	1	1	1	1	1	1	1	7
miR-570-3p	*RFX3*	1	1	1	1	1	1	1	7
miR-570-3p	*RORA*	1	1	1	1	1	1	1	7
miR-570-3p	*RPS6KA3*	1	1	1	1	1	1	1	7
miR-570-3p	*ATXN7*	1	1	1	1	1	1	1	7
miR-570-3p	*ITSN1*	1	1	1	1	1	1	1	7
miR-570-3p	*SNTB2*	1	1	1	1	1	1	1	7
miR-570-3p	*SRPK1*	1	1	1	1	1	1	1	7
miR-570-3p	*TROVE2*	1	1	1	1	1	1	1	7

Table S1: Continued

miRNA	Gene	miRWalk	Microt4	miRanda	miRMap	RNA22	RNAhybrid	Targetscan	SUM
miR-570-3p	*SSR1*	1	1	1	1	1	1	1	7
miR-570-3p	*ST13*	1	1	1	1	1	1	1	7
miR-570-3p	*STK4*	1	1	1	1	1	1	1	7
miR-570-3p	*GCFC2*	1	1	1	1	1	1	1	7
miR-570-3p	*TCF12*	1	1	1	1	1	1	1	7
miR-570-3p	*TRAPPC10*	1	1	1	1	1	1	1	7
miR-570-3p	*TPD52*	1	1	1	1	1	1	1	7
miR-570-3p	*TPM2*	1	1	1	1	1	1	1	7
miR-570-3p	*TRPS1*	1	1	1	1	1	1	1	7
miR-570-3p	*UBP1*	1	1	1	1	1	1	1	7
miR-570-3p	*NRIP1*	1	1	1	1	1	1	1	7
miR-570-3p	*FZD4*	1	1	1	1	1	1	1	7
miR-570-3p	*HIST2H2BE*	1	1	1	1	1	1	1	7
miR-570-3p	*TNKS*	1	1	1	1	1	1	1	7
miR-570-3p	*NRP1*	1	1	1	1	1	1	1	7
miR-570-3p	*ATG12*	1	1	1	1	1	1	1	7
miR-570-3p	*REPS2*	1	1	1	1	1	1	1	7
miR-570-3p	*VAPA*	1	1	1	1	1	1	1	7
miR-570-3p	*DLGAP1*	1	1	1	1	1	1	1	7
miR-570-3p	*BAG4*	1	1	1	1	1	1	1	7
miR-570-3p	*LRRC14*	1	1	1	1	1	1	1	7
miR-570-3p	*HDAC4*	1	1	1	1	1	1	1	7
miR-570-3p	*BCLAF1*	1	1	1	1	1	1	1	7
miR-570-3p	*ZBTB39*	1	1	1	1	1	1	1	7
miR-570-3p	*SRGAP3*	1	1	1	1	1	1	1	7
miR-570-3p	*PTBP3*	1	1	1	1	1	1	1	7
miR-570-3p	*AKT3*	1	1	1	1	1	1	1	7
miR-570-3p	*TSPAN3*	1	1	1	1	1	1	1	7
miR-570-3p	*OPTN*	1	1	1	1	1	1	1	7
miR-570-3p	*HMG20A*	1	1	1	1	1	1	1	7
miR-570-3p	*SYNPO*	1	1	1	1	1	1	1	7
miR-570-3p	*RRAS2*	1	1	1	1	1	1	1	7
miR-570-3p	*IKZF2*	1	1	1	1	1	1	1	7
miR-570-3p	*MTF2*	1	1	1	1	1	1	1	7
miR-570-3p	*COBLL1*	1	1	1	1	1	1	1	7
miR-570-3p	*ZNF507*	1	1	1	1	1	1	1	7
miR-570-3p	*PDS5B*	1	1	1	1	1	1	1	7
miR-570-3p	*TBC1D2B*	1	1	1	1	1	1	1	7
miR-570-3p	*GSE1*	1	1	1	1	1	1	1	7
miR-570-3p	*WSCD1*	1	1	1	1	1	1	1	7

Table S1: Continued

miRNA	Gene	miRWalk	Microt4	miRanda	miRMap	RNA22	RNAhybrid	Targetscan	SUM
miR-570-3p	*CRTC1*	1	1	1	1	1	1	1	7
miR-570-3p	*LRRC8B*	1	1	1	1	1	1	1	7
miR-570-3p	*ZNF451*	1	1	1	1	1	1	1	7
miR-570-3p	*ZBTB20*	1	1	1	1	1	1	1	7
miR-570-3p	*RBMS3*	1	1	1	1	1	1	1	7
miR-570-3p	*KCNMB4*	1	1	1	1	1	1	1	7
miR-570-3p	*ZBTB44*	1	1	1	1	1	1	1	7
miR-570-3p	*TMOD2*	1	1	1	1	1	1	1	7
miR-570-3p	*TRA2A*	1	1	1	1	1	1	1	7
miR-570-3p	*UBIAD1*	1	1	1	1	1	1	1	7
miR-570-3p	*RNF141*	1	1	1	1	1	1	1	7
miR-570-3p	*DCTN4*	1	1	1	1	1	1	1	7
miR-570-3p	*SS18L2*	1	1	1	1	1	1	1	7
miR-570-3p	*FAM53C*	1	1	1	1	1	1	1	7
miR-570-3p	*DACT1*	1	1	1	1	1	1	1	7
miR-570-3p	*RAPGEF6*	1	1	1	1	1	1	1	7
miR-570-3p	*RSF1*	1	1	1	1	1	1	1	7
miR-570-3p	*SNRK*	1	1	1	1	1	1	1	7
miR-570-3p	*COMMD4*	1	1	1	1	1	1	1	7
miR-570-3p	*FAM120C*	1	1	1	1	1	1	1	7
miR-570-3p	*STRBP*	1	1	1	1	1	1	1	7
miR-570-3p	*MBNL3*	1	1	1	1	1	1	1	7
miR-570-3p	*EIF5A2*	1	1	1	1	1	1	1	7
miR-570-3p	*SMARCAD1*	1	1	1	1	1	1	1	7
miR-570-3p	*PELI1*	1	1	1	1	1	1	1	7
miR-570-3p	*HEG1*	1	1	1	1	1	1	1	7
miR-570-3p	*CGN*	1	1	1	1	1	1	1	7
miR-570-3p	*TAOK1*	1	1	1	1	1	1	1	7
miR-570-3p	*STIM2*	1	1	1	1	1	1	1	7
miR-570-3p	*NYAP2*	1	1	1	1	1	1	1	7
miR-570-3p	*ALS2*	1	1	1	1	1	1	1	7
miR-570-3p	*USP37*	1	1	1	1	1	1	1	7
miR-570-3p	*TRAK2*	1	1	1	1	1	1	1	7
miR-570-3p	*ARSJ*	1	1	1	1	1	1	1	7
miR-570-3p	*PEAK1*	1	1	1	1	1	1	1	7
miR-570-3p	*SRCIN1*	1	1	1	1	1	1	1	7
miR-570-3p	*NUAK2*	1	1	1	1	1	1	1	7
miR-570-3p	*APH1B*	1	1	1	1	1	1	1	7
miR-570-3p	*SPRTN*	1	1	1	1	1	1	1	7
miR-570-3p	*KATNAL1*	1	1	1	1	1	1	1	7

Table S1: Continued

miRNA	Gene	miRWalk	Microt4	miRanda	miRMap	RNA22	RNAhybrid	Targetscan	SUM
miR-570-3p	*NFATC2IP*	1	1	1	1	1	1	1	7
miR-570-3p	*NAV1*	1	1	1	1	1	1	1	7
miR-570-3p	*TP53INP1*	1	1	1	1	1	1	1	7
miR-570-3p	*C19orf47*	1	1	1	1	1	1	1	7
miR-570-3p	*TCEANC2*	1	1	1	1	1	1	1	7
miR-570-3p	*CPEB2*	1	1	1	1	1	1	1	7
miR-570-3p	*FAM199X*	1	1	1	1	1	1	1	7
miR-570-3p	*C20orf112*	1	1	1	1	1	1	1	7
miR-570-3p	*NRSN1*	1	1	1	1	1	1	1	7
miR-570-3p	*RC3H1*	1	1	1	1	1	1	1	7
miR-570-3p	*FAM117B*	1	1	1	1	1	1	1	7
miR-570-3p	*AMOTL1*	1	1	1	1	1	1	1	7
miR-570-3p	*ZXDB*	1	1	1	1	1	1	1	7
miR-570-3p	*PIKFYVE*	1	1	1	1	1	1	1	7
miR-570-3p	*AMER2*	1	1	1	1	1	1	1	7
miR-570-3p	*SEMA3D*	1	1	1	1	1	1	1	7
miR-570-3p	*SLC17A8*	1	1	1	1	1	1	1	7
miR-570-3p	*GXYLT1*	1	1	1	1	1	1	1	7
miR-570-3p	*ZNF445*	1	1	1	1	1	1	1	7
miR-570-3p	*YPEL2*	1	1	1	1	1	1	1	7
miR-570-3p	*BEND4*	1	1	1	1	1	1	1	7
miR-570-3p	*ZBTB34*	1	1	1	1	1	1	1	7
miR-570-3p	*PSAPL1*	1	1	1	1	1	1	1	7
miR-570-3p	***CD44***	0	1	1	1	1	1	1	**6**
miR-570-3p	*ABL2*	1	1	1	1	0	1	1	6
miR-570-3p	*ACADSB*	1	1	1	1	0	1	1	6
miR-570-3p	*ACOX1*	1	1	1	1	0	1	1	6
miR-570-3p	*ACVR1*	1	1	1	1	0	1	1	6
miR-570-3p	*ADH5*	0	1	1	1	1	1	1	6
miR-570-3p	*AP1G1*	1	1	1	1	0	1	1	6
miR-570-3p	*AK2*	1	1	1	1	0	1	1	6
miR-570-3p	*BIN1*	1	1	1	1	0	1	1	6
miR-570-3p	*APBB2*	1	1	1	1	0	1	1	6
miR-570-3p	*RND3*	1	1	1	1	0	1	1	6
miR-570-3p	*ARNT*	1	1	1	1	0	1	1	6
miR-570-3p	*ASTN1*	1	1	1	1	0	1	1	6
miR-570-3p	*RERE*	1	1	1	1	0	1	1	6
miR-570-3p	*ATP1B1*	1	1	1	1	0	1	1	6
miR-570-3p	*ATP1B2*	1	1	1	1	0	1	1	6
miR-570-3p	*ATP2A2*	1	1	1	1	0	1	1	6

Table S1: Continued

miRNA	Gene	miRWalk	Microt4	miRanda	miRMap	RNA22	RNAhybrid	Targetscan	SUM
miR-570-3p	*ATP2B1*	1	1	1	1	0	1	1	6
miR-570-3p	*ATP2B2*	1	1	1	1	0	1	1	6
miR-570-3p	*ATP2B3*	1	1	1	1	0	1	1	6
miR-570-3p	*ATP2B3*	1	1	1	1	0	1	1	6
miR-570-3p	*ATP2B4*	1	1	1	1	0	1	1	6
miR-570-3p	*ALDH7A1*	1	1	1	1	0	1	1	6
miR-570-3p	*ALDH7A1*	1	1	1	1	0	1	1	6
miR-570-3p	*ATP6V1B2*	1	1	1	1	0	1	1	6
miR-570-3p	*ATP7A*	1	1	1	1	0	1	1	6
miR-570-3p	*ATP7A*	1	1	1	1	0	1	1	6
miR-570-3p	*ATP7B*	1	1	1	1	0	1	1	6
miR-570-3p	*BACH1*	1	1	1	1	0	1	1	6
miR-570-3p	*BAG1*	1	1	1	1	0	1	1	6
miR-570-3p	*BAG1*	1	1	1	1	0	1	1	6
miR-570-3p	*BAI3*	1	1	1	1	0	1	1	6
miR-570-3p	*BCL2L2*	1	1	1	1	0	1	1	6
miR-570-3p	*BCL9*	1	1	1	1	0	1	1	6
miR-570-3p	*BICD1*	1	1	1	1	0	1	1	6
miR-570-3p	*BIK*	1	1	1	1	0	1	1	6
miR-570-3p	*BNC1*	1	1	1	1	0	1	1	6
miR-570-3p	*BMP2*	1	1	1	1	0	1	1	6
miR-570-3p	*BMP3*	1	1	1	1	0	1	1	6
miR-570-3p	*BMP7*	1	1	1	1	0	1	1	6
miR-570-3p	*BMPR1B*	1	1	1	1	0	1	1	6
miR-570-3p	*BMPR2*	1	1	1	1	0	1	1	6
miR-570-3p	*KLF9*	1	1	1	1	0	1	1	6
miR-570-3p	*OSGIN2*	1	1	1	1	0	1	1	6
miR-570-3p	*MPPED2*	1	1	1	1	0	1	1	6
miR-570-3p	*CACNA1B*	1	1	1	1	0	1	1	6
miR-570-3p	*CACNA1E*	1	1	1	1	0	1	1	6
miR-570-3p	*CACNB4*	1	1	1	1	0	1	1	6
miR-570-3p	*CALB1*	1	1	1	1	0	1	1	6
miR-570-3p	*CALD1*	1	1	1	1	0	1	1	6
miR-570-3p	*CCND2*	1	1	1	1	0	1	1	6
miR-570-3p	*CD28*	1	1	1	1	0	1	1	6
miR-570-3p	*CIRBP*	1	1	0	1	1	1	1	6
miR-570-3p	*CLCN3*	1	1	1	1	0	1	1	6
miR-570-3p	*CMKLR1*	1	1	1	1	0	1	1	6
miR-570-3p	*COL4A4*	1	1	1	1	0	1	1	6
miR-570-3p	*COL8A2*	1	1	1	1	0	1	1	6

Table S1: Continued

miRNA	Gene	miRWalk	Microt4	miRanda	miRMap	RNA22	RNAhybrid	Targetscan	SUM
miR-570-3p	*KLF6*	1	1	1	1	0	1	1	6
miR-570-3p	*CPD*	1	1	1	1	0	1	1	6
miR-570-3p	*CREBBP*	1	1	1	1	0	1	1	6
miR-570-3p	*CREBL2*	0	1	1	1	1	1	1	6
miR-570-3p	*CRKL*	1	1	1	1	0	1	1	6
miR-570-3p	*HAPLN1*	1	1	1	1	0	1	1	6
miR-570-3p	*CYLD*	1	1	1	1	0	1	1	6
miR-570-3p	*CYP26A1*	1	1	1	1	0	1	1	6
miR-570-3p	*DAB1*	1	1	1	1	0	1	1	6
miR-570-3p	*DAG1*	1	1	1	1	0	1	1	6
miR-570-3p	*DGKB*	1	1	1	1	0	1	1	6
miR-570-3p	*DCX*	1	1	1	1	0	1	1	6
miR-570-3p	*DMXL1*	0	1	1	1	1	1	1	6
miR-570-3p	*DLX2*	1	1	1	1	0	1	1	6
miR-570-3p	*DMD*	0	1	1	1	1	1	1	6
miR-570-3p	*DPYSL3*	1	1	1	1	0	1	1	6
miR-570-3p	*DSC3*	1	1	1	1	0	1	1	6
miR-570-3p	*DTNA*	1	1	1	1	0	1	1	6
miR-570-3p	*DUSP4*	0	1	1	1	1	1	1	6
miR-570-3p	*DUSP8*	1	1	1	1	0	1	1	6
miR-570-3p	*DVL3*	1	1	1	1	0	1	1	6
miR-570-3p	*EFNA5*	1	1	1	1	0	1	1	6
miR-570-3p	*EIF4G2*	1	1	1	1	0	1	1	6
miR-570-3p	*SERPINB1*	1	1	1	1	0	1	1	6
miR-570-3p	*ELAVL2*	1	1	1	1	0	1	1	6
miR-570-3p	*ELAVL1*	1	1	1	1	0	1	1	6
miR-570-3p	*ELK4*	0	1	1	1	1	1	1	6
miR-570-3p	*EPHA3*	1	1	1	1	0	1	1	6
miR-570-3p	*EPHA5*	1	1	1	1	0	1	1	6
miR-570-3p	*EPHA7*	1	1	1	1	0	1	1	6
miR-570-3p	*EYA4*	1	1	1	1	0	1	1	6
miR-570-3p	*ERG*	1	1	1	1	0	1	1	6
miR-570-3p	*ESR1*	1	1	1	1	0	1	1	6
miR-570-3p	*ETS1*	1	1	1	1	0	1	1	6
miR-570-3p	*ETS2*	0	1	1	1	1	1	1	6
miR-570-3p	*ETV5*	0	1	1	1	1	1	1	6
miR-570-3p	*ETV6*	1	1	1	1	0	1	1	6
miR-570-3p	*EYA1*	1	1	1	1	0	1	1	6
miR-570-3p	*EZH1*	1	1	1	1	0	1	1	6
miR-570-3p	*FGF2*	1	1	1	1	0	1	1	6

Table S1: Continued

miRNA	Gene	miRWalk	Microt4	miRanda	miRMap	RNA22	RNAhybrid	Targetscan	SUM
miR-570-3p	*FOXO3*	0	1	1	1	1	1	1	6
miR-570-3p	*FMR1*	1	1	1	1	0	1	1	6
miR-570-3p	*AFF2*	1	1	1	1	0	1	1	6
miR-570-3p	*FUT1*	1	1	1	1	0	1	1	6
miR-570-3p	*FUT1*	1	1	1	1	0	1	1	6
miR-570-3p	*KDSR*	1	1	1	1	0	1	1	6
miR-570-3p	*GABPA*	1	1	1	1	0	1	1	6
miR-570-3p	*GABRA1*	1	1	1	1	0	1	1	6
miR-570-3p	*GABRA4*	0	1	1	1	1	1	1	6
miR-570-3p	*GABRB2*	0	1	1	1	1	1	1	6
miR-570-3p	*GANC*	1	1	1	1	0	1	1	6
miR-570-3p	*NR6A1*	1	1	1	1	0	1	1	6
miR-570-3p	*GDNF*	1	1	1	1	0	1	1	6
miR-570-3p	*GFRA1*	1	1	1	1	0	1	1	6
miR-570-3p	*GPC3*	1	1	1	1	0	1	1	6
miR-570-3p	*GLS*	1	1	1	1	0	1	1	6
miR-570-3p	*GNAI1*	1	1	1	1	0	1	1	6
miR-570-3p	*GNAL*	0	1	1	1	1	1	1	6
miR-570-3p	*GNG4*	0	1	1	1	1	1	1	6
miR-570-3p	*GPD2*	1	1	1	1	0	1	1	6
miR-570-3p	*GPM6A*	0	1	1	1	1	1	1	6
miR-570-3p	*RAPGEF1*	1	1	1	1	0	1	1	6
miR-570-3p	*GRIA3*	0	1	1	1	1	1	1	6
miR-570-3p	*GRIA3*	0	1	1	1	1	1	1	6
miR-570-3p	*GRIK2*	1	1	1	1	0	1	1	6
miR-570-3p	*GRM1*	1	1	1	1	0	1	1	6
miR-570-3p	*GRM7*	1	1	1	1	0	1	1	6
miR-570-3p	*GRSF1*	1	1	1	1	0	1	1	6
miR-570-3p	*GSPT1*	1	1	1	1	0	1	1	6
miR-570-3p	*GUCY1A2*	1	1	1	1	0	1	1	6
miR-570-3p	*HAS3*	1	1	1	1	0	1	1	6
miR-570-3p	*HCFC1*	1	1	1	1	0	1	1	6
miR-570-3p	*HCFC1*	1	1	1	1	0	1	1	6
miR-570-3p	*HDAC2*	1	1	1	1	0	1	1	6
miR-570-3p	*HGF*	0	1	1	1	1	1	1	6
miR-570-3p	*HIP1*	1	1	1	1	0	1	1	6
miR-570-3p	*HIP1*	1	1	1	1	0	1	1	6
miR-570-3p	*UBE2K*	1	1	1	1	0	1	1	6
miR-570-3p	*HLF*	1	1	1	1	0	1	1	6
miR-570-3p	*HMGCS1*	1	1	1	1	0	1	1	6

Table S1: Continued

miRNA	Gene	miRWalk	Microt4	miRanda	miRMap	RNA22	RNAhybrid	Targetscan	SUM
miR-570-3p	*HNF4G*	1	1	1	1	0	1	1	6
miR-570-3p	*HNRNPA2B1*	1	1	1	1	0	1	1	6
miR-570-3p	*FOXN2*	1	1	1	1	0	1	1	6
miR-570-3p	*HTR1E*	1	1	1	1	0	1	1	6
miR-570-3p	*IFNAR1*	1	1	1	1	0	1	1	6
miR-570-3p	*IGF1R*	0	1	1	1	1	1	1	6
miR-570-3p	*IGFBP5*	1	1	1	1	0	1	1	6
miR-570-3p	*RBPJ*	1	1	1	1	0	1	1	6
miR-570-3p	*IL1RAP*	1	1	1	1	0	1	1	6
miR-570-3p	*IL10*	1	1	1	1	0	1	1	6
miR-570-3p	*INSR*	1	1	1	1	0	1	1	6
miR-570-3p	*ISL1*	1	1	1	1	0	1	1	6
miR-570-3p	*ITGB3*	1	1	1	1	0	1	1	6
miR-570-3p	*ITPR2*	1	1	1	1	0	1	1	6
miR-570-3p	*JAK2*	1	1	1	1	0	1	1	6
miR-570-3p	*KCNJ3*	1	1	1	1	0	1	1	6
miR-570-3p	*KCNJ13*	1	1	1	1	0	1	1	6
miR-570-3p	*KCNMA1*	1	1	1	1	0	1	1	6
miR-570-3p	*KIF5B*	1	1	1	1	0	1	1	6
miR-570-3p	*KPNA3*	1	1	1	1	0	1	1	6
miR-570-3p	*KPNA4*	1	1	1	1	0	1	1	6
miR-570-3p	*IPO5*	0	1	1	1	1	1	1	6
miR-570-3p	*LAMC1*	1	1	1	1	0	1	1	6
miR-570-3p	*LAMP2*	0	1	1	1	1	1	1	6
miR-570-3p	*LIFR*	1	1	1	1	0	1	1	6
miR-570-3p	*LMX1A*	0	1	1	1	1	1	1	6
miR-570-3p	*LPP*	0	1	1	1	1	1	1	6
miR-570-3p	*MXD1*	0	1	1	1	1	1	1	6
miR-570-3p	*SMAD3*	1	1	1	1	0	1	1	6
miR-570-3p	*SMAD4*	1	1	1	1	0	1	1	6
miR-570-3p	*MAF*	1	1	1	1	0	1	1	6
miR-570-3p	*MBL2*	0	1	1	1	1	1	1	6
miR-570-3p	*MBNL1*	0	1	1	1	1	1	1	6
miR-570-3p	*MECP2*	1	1	1	1	0	1	1	6
miR-570-3p	*MECP2*	1	1	1	1	0	1	1	6
miR-570-3p	*MEF2A*	1	1	1	1	0	1	1	6
miR-570-3p	*MEIS2*	1	1	1	1	0	1	1	6
miR-570-3p	*MAP3K1*	1	1	1	1	0	1	1	6
miR-570-3p	*MFAP1*	1	1	1	1	0	1	1	6
miR-570-3p	*MFAP3*	1	1	1	1	0	1	1	6

Table S1: Continued

miRNA	Gene	miRWalk	Microt4	miRanda	miRMap	RNA22	RNAhybrid	Targetscan	SUM
miR-570-3p	*MGAT2*	1	1	1	1	0	1	1	6
miR-570-3p	*MGAT5*	1	1	1	1	0	1	1	6
miR-570-3p	*ATXN3*	1	1	1	1	0	1	1	6
miR-570-3p	*AFF1*	1	1	1	1	0	1	1	6
miR-570-3p	*AFF1*	1	1	1	1	0	1	1	6
miR-570-3p	*MMP1*	1	1	1	1	0	1	1	6
miR-570-3p	*MMP8*	1	1	1	1	0	1	1	6
miR-570-3p	*MN1*	1	1	1	1	0	1	1	6
miR-570-3p	*MYO1B*	1	1	1	1	0	1	1	6
miR-570-3p	*MSX2*	1	1	1	1	0	1	1	6
miR-570-3p	*MTAP*	1	1	1	1	0	1	1	6
miR-570-3p	*MXI1*	1	1	1	1	0	1	1	6
miR-570-3p	*MYH9*	1	1	1	1	0	1	1	6
miR-570-3p	*MYO1C*	1	1	1	1	0	1	1	6
miR-570-3p	*MYO6*	1	1	1	1	0	1	1	6
miR-570-3p	*MYO9A*	1	1	1	1	0	1	1	6
miR-570-3p	*PPP1R12A*	1	1	1	1	0	1	1	6
miR-570-3p	*PPP1R12B*	1	1	1	1	0	1	1	6
miR-570-3p	*MYT1*	1	1	1	1	0	1	1	6
miR-570-3p	*NAP1L2*	1	1	1	1	0	1	1	6
miR-570-3p	*NAP1L4*	1	1	1	1	0	1	1	6
miR-570-3p	*NBN*	1	1	1	1	0	1	1	6
miR-570-3p	*NCAM1*	1	1	1	1	0	1	1	6
miR-570-3p	*NCK1*	1	1	1	1	0	1	1	6
miR-570-3p	*NDUFA4*	1	1	1	1	0	1	1	6
miR-570-3p	*NDUFA5*	1	1	1	1	0	1	1	6
miR-570-3p	*NDUFA10*	1	1	1	1	0	1	1	6
miR-570-3p	*Sep-02*	1	1	1	1	0	1	1	6
miR-570-3p	*NEDD9*	1	1	1	1	0	1	1	6
miR-570-3p	*NEFH*	1	1	1	1	0	1	1	6
miR-570-3p	*NEK1*	1	1	1	1	0	1	1	6
miR-570-3p	*NFATC1*	1	1	1	1	0	1	1	6
miR-570-3p	*NFIA*	1	1	1	1	0	1	1	6
miR-570-3p	*NFATC3*	1	1	1	1	0	1	1	6
miR-570-3p	*NFIB*	1	1	1	1	0	1	1	6
miR-570-3p	*NFIC*	1	1	1	1	0	1	1	6
miR-570-3p	*NFIX*	1	1	1	1	0	1	1	6
miR-570-3p	*NFYB*	1	1	1	1	0	1	1	6
miR-570-3p	*NHS*	1	1	1	1	0	1	1	6
miR-570-3p	*NOS1*	1	1	1	1	0	1	1	6

Table S1: Continued

miRNA	Gene	miRWalk	Microt4	miRanda	miRMap	RNA22	RNAhybrid	Targetscan	SUM
miR-570-3p	*CNOT4*	1	1	1	1	0	1	1	6
miR-570-3p	*NOTCH2*	1	1	1	1	0	1	1	6
miR-570-3p	*NPR3*	1	1	1	1	0	1	1	6
miR-570-3p	*NRAS*	1	1	1	1	0	1	1	6
miR-570-3p	*NTRK2*	1	1	1	1	0	1	1	6
miR-570-3p	*NTRK3*	1	1	1	1	0	1	1	6
miR-570-3p	*ROR1*	1	1	1	1	0	1	1	6
miR-570-3p	*NTS*	1	1	1	1	0	1	1	6
miR-570-3p	*OAZ2*	1	1	1	1	0	1	1	6
miR-570-3p	*OCRL*	1	1	1	1	0	1	1	6
miR-570-3p	*ODF2*	1	1	1	1	0	1	1	6
miR-570-3p	*OPA1*	1	1	1	1	0	1	1	6
miR-570-3p	*OPCML*	1	1	1	1	0	1	1	6
miR-570-3p	*OPHN1*	1	1	1	1	0	1	1	6
miR-570-3p	*OSM*	1	1	1	1	0	1	1	6
miR-570-3p	*FURIN*	1	1	1	1	0	1	1	6
miR-570-3p	*PAFAH1B1*	1	1	1	1	0	1	1	6
miR-570-3p	*PAFAH1B2*	1	1	1	1	0	1	1	6
miR-570-3p	*PAFAH2*	1	1	1	1	0	1	1	6
miR-570-3p	*PAK2*	1	1	1	1	0	1	1	6
miR-570-3p	*PAM*	1	1	1	1	0	1	1	6
miR-570-3p	*PAPPA*	1	1	1	1	0	1	1	6
miR-570-3p	*PAX2*	1	1	1	1	0	1	1	6
miR-570-3p	*PAX5*	1	1	1	1	0	1	1	6
miR-570-3p	*PDCL*	1	1	1	1	0	1	1	6
miR-570-3p	*PCCB*	1	1	1	1	0	1	1	6
miR-570-3p	*PCSK5*	1	1	1	1	0	1	1	6
miR-570-3p	*PDE1A*	1	1	1	1	0	1	1	6
miR-570-3p	*PDE4B*	1	1	1	1	0	1	1	6
miR-570-3p	*PDE4D*	1	1	1	1	0	1	1	6
miR-570-3p	*PDE6A*	1	1	1	1	0	1	1	6
miR-570-3p	*PDE9A*	1	1	1	1	0	1	1	6
miR-570-3p	*PDHA1*	1	1	1	1	0	1	1	6
miR-570-3p	*PDPK1*	1	1	1	1	0	1	1	6
miR-570-3p	*CDK14*	1	1	1	1	0	1	1	6
miR-570-3p	*PGGT1B*	1	1	1	1	0	1	1	6
miR-570-3p	*PGM5*	1	1	1	1	0	1	1	6
miR-570-3p	*PGR*	1	1	1	1	0	1	1	6
miR-570-3p	*PHF2*	1	1	1	1	0	1	1	6
miR-570-3p	*PIGA*	1	1	1	1	0	1	1	6

Table S1: Continued

miRNA	Gene	miRWalk	Microt4	miRanda	miRMap	RNA22	RNAhybrid	Targetscan	SUM
miR-570-3p	*PIK3C2A*	1	1	1	1	0	1	1	6
miR-570-3p	*PITPNA*	1	1	1	1	0	1	1	6
miR-570-3p	*PKNOX1*	1	1	1	1	0	1	1	6
miR-570-3p	*PLAG1*	1	1	1	1	0	1	1	6
miR-570-3p	*PLAGL2*	1	1	1	1	0	1	1	6
miR-570-3p	*PLAT*	1	1	1	1	0	1	1	6
miR-570-3p	*PLD1*	1	1	1	1	0	1	1	6
miR-570-3p	*PLP1*	1	1	1	1	0	1	1	6
miR-570-3p	*PLS1*	1	1	1	1	0	1	1	6
miR-570-3p	*PRRX1*	1	1	1	1	0	1	1	6
miR-570-3p	*PNN*	1	1	1	1	0	1	1	6
miR-570-3p	*UBL3*	1	1	1	1	0	1	1	6
miR-570-3p	*PODXL*	1	1	1	1	0	1	1	6
miR-570-3p	*POR*	1	1	1	1	0	1	1	6
miR-570-3p	*POU2F1*	1	1	1	1	0	1	1	6
miR-570-3p	*PPEF2*	1	1	1	1	0	1	1	6
miR-570-3p	*PPAT*	1	1	1	1	0	1	1	6
miR-570-3p	*PPIA*	1	1	1	1	0	1	1	6
miR-570-3p	*PPM1A*	1	1	1	1	0	1	1	6
miR-570-3p	*PPM1B*	1	1	1	1	0	1	1	6
miR-570-3p	*PPM1G*	1	1	1	1	0	1	1	6
miR-570-3p	*PPP1CB*	1	1	1	1	0	1	1	6
miR-570-3p	*PPP1R2*	1	1	1	1	0	1	1	6
miR-570-3p	*PPP1R3D*	1	1	1	1	0	1	1	6
miR-570-3p	*PPP1R10*	1	1	1	1	0	1	1	6
miR-570-3p	*PPP2R5C*	1	1	1	1	0	1	1	6
miR-570-3p	*PPP3CA*	1	1	1	1	0	1	1	6
miR-570-3p	*PPP3CB*	1	1	1	1	0	1	1	6
miR-570-3p	*PRKAA1*	1	1	1	1	0	1	1	6
miR-570-3p	*PKIA*	1	1	1	1	0	1	1	6
miR-570-3p	*PKIB*	1	1	1	1	0	1	1	6
miR-570-3p	*PRKAR2A*	1	1	1	1	0	1	1	6
miR-570-3p	*PRKCA*	1	1	1	1	0	1	1	6
miR-570-3p	*PRKCB*	1	1	1	1	0	1	1	6
miR-570-3p	*PRKG1*	1	1	1	1	0	1	1	6
miR-570-3p	*MAPK9*	1	1	1	1	0	1	1	6
miR-570-3p	*MAPK10*	1	1	1	1	0	1	1	6
miR-570-3p	*MAPK10*	1	1	1	1	0	1	1	6
miR-570-3p	*PRLR*	1	1	1	1	0	1	1	6
miR-570-3p	*PROX1*	1	1	1	1	0	1	1	6

Table S1: Continued

miRNA	Gene	miRWalk	Microt4	miRanda	miRMap	RNA22	RNAhybrid	Targetscan	SUM
miR-570-3p	*PRRG1*	1	1	1	1	0	1	1	6
miR-570-3p	*MASP1*	1	1	1	1	0	1	1	6
miR-570-3p	*PSEN1*	1	1	1	1	0	1	1	6
miR-570-3p	*PSMC6*	1	1	1	1	0	1	1	6
miR-570-3p	*PSMD10*	1	1	1	1	0	1	1	6
miR-570-3p	*PSMD11*	1	1	1	1	0	1	1	6
miR-570-3p	*PTCH1*	1	1	1	1	0	1	1	6
miR-570-3p	*PTGFRN*	1	1	1	1	0	1	1	6
miR-570-3p	*PTGS2*	1	1	1	1	0	1	1	6
miR-570-3p	*TWF1*	1	1	1	1	0	1	1	6
miR-570-3p	*PTN*	1	1	1	1	0	1	1	6
miR-570-3p	*PTPN4*	1	1	1	1	0	1	1	6
miR-570-3p	*PTPN11*	1	1	1	1	0	1	1	6
miR-570-3p	*PTPN14*	1	1	1	1	0	1	1	6
miR-570-3p	*PTPRB*	1	1	1	1	0	1	1	6
miR-570-3p	*PTPRD*	1	1	1	1	0	1	1	6
miR-570-3p	*PTPRJ*	1	1	0	1	1	1	1	6
miR-570-3p	*PTPRO*	1	1	1	1	0	1	1	6
miR-570-3p	*PTPRZ1*	1	1	1	1	0	1	1	6
miR-570-3p	*RAD1*	1	1	1	1	0	1	1	6
miR-570-3p	*PURB*	1	1	1	1	0	1	1	6
miR-570-3p	*PVRL1*	1	1	1	1	0	1	1	6
miR-570-3p	*PXN*	1	1	1	1	0	1	1	6
miR-570-3p	*QDPR*	1	1	1	1	0	1	1	6
miR-570-3p	*RAB2A*	1	1	1	1	0	1	1	6
miR-570-3p	*RAB3B*	1	1	1	1	0	1	1	6
miR-570-3p	*RAB4A*	1	1	1	1	0	1	1	6
miR-570-3p	*RAC1*	1	1	1	1	0	1	1	6
miR-570-3p	*RAD23B*	1	1	1	1	0	1	1	6
miR-570-3p	*RAF1*	1	1	1	1	0	1	1	6
miR-570-3p	*RAG1*	1	1	1	1	0	1	1	6
miR-570-3p	*RAP1GDS1*	1	1	1	1	0	1	1	6
miR-570-3p	*RASA2*	1	1	1	1	0	1	1	6
miR-570-3p	*RB1*	1	1	1	1	0	1	1	6
miR-570-3p	*RBBP4*	1	1	1	1	0	1	1	6
miR-570-3p	*RBBP5*	1	1	1	1	0	1	1	6
miR-570-3p	*RDX*	1	1	1	1	0	1	1	6
miR-570-3p	*UPF1*	1	1	1	1	0	1	1	6
miR-570-3p	*RFX1*	1	1	1	1	0	1	1	6
miR-570-3p	*RGS16*	1	1	1	1	0	1	1	6

Table S1: Continued

miRNA	Gene	miRWalk	Microt4	miRanda	miRMap	RNA22	RNAhybrid	Targetscan	SUM
miR-570-3p	*RHD*	1	1	1	1	0	1	1	6
miR-570-3p	*BRD2*	1	1	1	1	0	1	1	6
miR-570-3p	*ABCE1*	1	1	1	1	0	1	1	6
miR-570-3p	*ROBO1*	1	1	1	1	0	1	1	6
miR-570-3p	*ROBO2*	1	1	1	1	0	1	1	6
miR-570-3p	*ROCK1*	1	1	1	0	1	1	1	6
miR-570-3p	*RPS6KA2*	1	1	1	1	0	1	1	6
miR-570-3p	*RPS6KB1*	1	1	1	1	0	1	1	6
miR-570-3p	*CLIP1*	1	1	1	1	0	1	1	6
miR-570-3p	*CLIP1*	1	1	1	1	0	1	1	6
miR-570-3p	*RYR2*	1	1	1	1	0	1	1	6
miR-570-3p	*SATB1*	1	1	1	1	0	1	1	6
miR-570-3p	*ATXN1*	1	1	1	1	0	1	1	6
miR-570-3p	*ATXN2*	1	1	1	1	0	1	1	6
miR-570-3p	*SCN9A*	1	1	1	1	0	1	1	6
miR-570-3p	*SDC2*	1	1	1	1	0	1	1	6
miR-570-3p	*SEL1L*	1	1	1	1	0	1	1	6
miR-570-3p	*SFRP1*	1	1	1	1	0	1	1	6
miR-570-3p	*SFRP2*	1	1	1	1	0	1	1	6
miR-570-3p	*SRSF2*	1	1	1	1	0	1	1	6
miR-570-3p	*SRSF5*	1	1	1	1	0	1	1	6
miR-570-3p	*SRSF6*	1	1	1	1	0	1	1	6
miR-570-3p	*SGCD*	1	1	1	1	0	1	1	6
miR-570-3p	*ST8SIA1*	1	1	1	1	0	1	1	6
miR-570-3p	*SIM1*	1	1	1	1	0	1	1	6
miR-570-3p	*SKI*	1	1	1	1	0	1	1	6
miR-570-3p	*SKIL*	1	1	1	1	0	1	1	6
miR-570-3p	*SKP2*	1	1	1	1	0	1	1	6
miR-570-3p	*SLA*	1	1	1	1	0	1	1	6
miR-570-3p	*SLC1A2*	1	1	1	1	0	1	1	6
miR-570-3p	*SLC1A4*	1	1	1	1	0	1	1	6
miR-570-3p	*SLC5A3*	1	1	1	1	0	1	1	6
miR-570-3p	*SLC6A6*	1	1	1	1	0	1	1	6
miR-570-3p	*SLC8A1*	1	1	1	1	0	1	1	6
miR-570-3p	*SLC8A3*	1	1	1	1	0	1	1	6
miR-570-3p	*SLC18A2*	1	1	1	1	0	1	1	6
miR-570-3p	*SLC22A5*	1	1	1	1	0	1	1	6
miR-570-3p	*SIGLEC1*	1	1	1	1	0	1	1	6
miR-570-3p	*SORL1*	1	1	1	1	0	1	1	6
miR-570-3p	*SOS1*	1	1	1	1	0	1	1	6

Table S1: Continued

miRNA	Gene	miRWalk	Microt4	miRanda	miRMap	RNA22	RNAhybrid	Targetscan	SUM
miR-570-3p	*SOX1*	1	1	1	1	0	1	1	6
miR-570-3p	*SOX3*	1	1	1	1	0	1	1	6
miR-570-3p	*SOX5*	1	1	1	1	0	1	1	6
miR-570-3p	*SOX9*	1	1	1	1	0	1	1	6
miR-570-3p	*SP1*	1	1	1	1	0	1	1	6
miR-570-3p	*SP3*	1	1	1	1	0	1	1	6
miR-570-3p	*SP4*	1	1	1	1	0	1	1	6
miR-570-3p	*SPAG1*	1	1	1	1	0	1	1	6
miR-570-3p	*SPTAN1*	1	1	1	1	0	1	1	6
miR-570-3p	*SRP9*	1	1	1	1	0	1	1	6
miR-570-3p	*SRP14*	1	1	1	1	0	1	1	6
miR-570-3p	*SRP54*	1	1	1	1	0	1	1	6
miR-570-3p	*SSTR3*	1	1	1	1	0	1	1	6
miR-570-3p	*HSPA13*	1	1	1	1	0	1	1	6
miR-570-3p	*STRN*	1	1	1	1	0	1	1	6
miR-570-3p	*STX3*	1	1	1	1	0	1	1	6
miR-570-3p	*MED22*	1	1	1	1	0	1	1	6
miR-570-3p	*VAMP1*	1	1	1	1	0	1	1	6
miR-570-3p	*SYK*	1	1	1	1	0	1	1	6
miR-570-3p	*SYT1*	1	1	1	1	0	1	1	6
miR-570-3p	*TAC1*	1	1	1	1	0	1	1	6
miR-570-3p	*TAF4B*	1	1	1	1	0	1	1	6
miR-570-3p	*TAF11*	1	1	1	1	0	1	1	6
miR-570-3p	*TBCA*	1	1	1	1	0	1	1	6
miR-570-3p	*TBCC*	0	1	1	1	1	1	1	6
miR-570-3p	*TCF4*	1	1	1	1	0	1	1	6
miR-570-3p	*TBX3*	1	1	1	1	0	1	1	6
miR-570-3p	*HNF1B*	1	1	1	1	0	1	1	6
miR-570-3p	*TCF7L2*	1	1	1	1	0	1	1	6
miR-570-3p	*TCF7L2*	1	1	1	1	0	1	1	6
miR-570-3p	*TCF20*	1	1	1	1	0	1	1	6
miR-570-3p	*TCF21*	1	1	1	1	0	1	1	6
miR-570-3p	*TCTA*	1	1	1	1	0	1	1	6
miR-570-3p	*TEAD1*	1	1	1	1	0	1	1	6
miR-570-3p	*TERF2*	1	1	1	1	0	1	1	6
miR-570-3p	*TFAM*	1	1	1	1	0	1	1	6
miR-570-3p	*TFAP2B*	1	1	1	1	0	1	1	6
miR-570-3p	*TFDP1*	1	1	1	1	0	1	1	6
miR-570-3p	*TFRC*	1	1	1	1	0	1	1	6
miR-570-3p	*TGFA*	1	1	1	1	0	1	1	6

Table S1: Continued

miRNA	Gene	miRWalk	Microt4	miRanda	miRMap	RNA22	RNAhybrid	Targetscan	SUM
miR-570-3p	*TGFA*	1	1	1	1	0	1	1	6
miR-570-3p	*TGFBR1*	1	1	1	1	0	1	1	6
miR-570-3p	*THBD*	1	1	1	1	0	1	1	6
miR-570-3p	*THRB*	1	1	1	1	0	1	1	6
miR-570-3p	*KLF10*	1	1	1	1	0	1	1	6
miR-570-3p	*TIA1*	1	1	1	1	0	1	1	6
miR-570-3p	*TIMP2*	1	1	1	1	0	1	1	6
miR-570-3p	*TIMP3*	1	1	1	1	0	1	1	6
miR-570-3p	*TIMP4*	1	1	1	1	0	1	1	6
miR-570-3p	*TNF*	1	1	1	1	0	1	1	6
miR-570-3p	*TNFAIP3*	1	1	1	1	0	1	1	6
miR-570-3p	*TOP2A*	1	1	1	1	0	1	1	6
miR-570-3p	*TPM3*	1	1	1	1	0	1	1	6
miR-570-3p	*TPP2*	1	1	1	1	0	1	1	6
miR-570-3p	*TRAF3*	1	1	1	1	0	1	1	6
miR-570-3p	*TTK*	1	1	1	1	0	1	1	6
miR-570-3p	*TTR*	1	1	1	1	0	1	1	6
miR-570-3p	*TUFT1*	1	1	1	1	0	1	1	6
miR-570-3p	*UBE2E1*	1	1	1	1	0	1	1	6
miR-570-3p	*UBE2H*	1	1	1	1	0	1	1	6
miR-570-3p	*SUMO1*	1	1	1	1	0	1	1	6
miR-570-3p	*UBTF*	1	1	1	1	0	1	1	6
miR-570-3p	*UGT8*	1	1	1	1	0	1	1	6
miR-570-3p	*UTRN*	1	1	1	1	0	1	1	6
miR-570-3p	*KDM6A*	1	1	1	1	0	1	1	6
miR-570-3p	*EIF4H*	1	1	1	1	0	1	1	6
miR-570-3p	*WEE1*	1	1	1	1	0	1	1	6
miR-570-3p	*WNT7B*	1	1	1	1	0	1	1	6
miR-570-3p	*WRN*	1	1	1	1	0	1	1	6
miR-570-3p	*XPO1*	1	1	1	1	0	1	1	6
miR-570-3p	*XRCC4*	1	1	1	1	0	1	1	6
miR-570-3p	*YES1*	1	1	1	1	0	1	1	6
miR-570-3p	*YWHAG*	1	1	1	1	0	1	1	6
miR-570-3p	*YWHAZ*	1	1	1	1	0	1	1	6
miR-570-3p	*ZFX*	1	1	1	1	0	1	1	6
miR-570-3p	*ZFY*	1	1	1	1	0	1	1	6
miR-570-3p	*ZIC1*	1	1	1	1	0	1	1	6
miR-570-3p	*ZIC3*	1	1	1	1	0	1	1	6
miR-570-3p	*ZNF3*	1	1	1	1	0	1	1	6
miR-570-3p	*ZNF12*	1	1	1	1	0	1	1	6

Table S1: Continued

miRNA	Gene	miRWalk	Microt4	miRanda	miRMap	RNA22	RNAhybrid	Targetscan	SUM
miR-570-3p	*ZNF22*	1	1	1	1	0	1	1	6
miR-570-3p	*ZNF24*	1	1	1	1	0	1	1	6
miR-570-3p	*ZNF35*	1	1	1	1	0	1	1	6
miR-570-3p	*ZNF74*	1	1	1	1	0	1	1	6
miR-570-3p	*ZNF84*	1	1	1	1	0	1	1	6
miR-570-3p	*ZNF148*	1	1	1	1	0	1	1	6
miR-570-3p	*ZSCAN9*	1	1	1	1	0	1	1	6
miR-570-3p	*ZFAND5*	1	1	1	1	0	1	1	6
miR-570-3p	*ZXDA*	1	1	1	1	0	1	1	6
miR-570-3p	*ST8SIA4*	1	1	1	1	0	1	1	6
miR-570-3p	*MAFK*	1	1	1	1	0	1	1	6
miR-570-3p	*FZD3*	1	1	1	1	0	1	1	6
miR-570-3p	*TUSC3*	1	1	1	1	0	1	1	6
miR-570-3p	*BRD3*	1	1	1	1	0	1	1	6
miR-570-3p	*NCOA4*	1	1	1	1	0	1	1	6
miR-570-3p	*PTP4A2*	1	1	1	1	0	1	1	6
miR-570-3p	*KMT2D*	1	1	1	1	0	1	1	6
miR-570-3p	*FXR1*	1	1	1	1	0	1	1	6
miR-570-3p	*HMGA2*	1	1	1	1	0	1	1	6
miR-570-3p	*RND2*	1	1	1	1	0	1	1	6
miR-570-3p	*NCOA3*	1	1	1	1	0	1	1	6
miR-570-3p	*DGCR14*	1	1	1	1	0	1	1	6
miR-570-3p	*FZD7*	1	1	1	1	0	1	1	6
miR-570-3p	*NSMAF*	1	1	1	1	0	1	1	6
miR-570-3p	*CUL3*	1	1	1	1	0	1	1	6
miR-570-3p	*CUL2*	1	1	1	1	0	1	1	6
miR-570-3p	*ATRN*	1	1	1	1	0	1	1	6
miR-570-3p	*SMARCA5*	1	1	1	1	0	1	1	6
miR-570-3p	*FKBP6*	1	1	1	1	0	1	1	6
miR-570-3p	*CDC42BPA*	1	1	1	1	0	1	1	6
miR-570-3p	*MAP4K3*	1	1	1	1	0	1	1	6
miR-570-3p	*GCM1*	1	1	1	1	0	1	1	6
miR-570-3p	*GAS7*	1	1	1	1	0	1	1	6
miR-570-3p	*DGKE*	1	1	1	1	0	1	1	6
miR-570-3p	*AGPS*	1	1	1	1	0	1	1	6
miR-570-3p	*CGGBP1*	1	1	1	1	0	1	1	6
miR-570-3p	*BHLHE40*	1	1	1	1	0	1	1	6
miR-570-3p	*PDLIM4*	1	1	1	1	0	1	1	6
miR-570-3p	*TNFSF11*	1	1	1	1	0	1	1	6
miR-570-3p	*STC2*	1	1	1	1	0	1	1	6

Table S1: Continued

miRNA	Gene	miRWalk	Microt4	miRanda	miRMap	RNA22	RNAhybrid	Targetscan	SUM
miR-570-3p	*PDE8B*	1	1	1	1	0	1	1	6
miR-570-3p	*TP63*	1	1	1	1	0	1	1	6
miR-570-3p	*PDE5A*	1	1	1	1	0	1	1	6
miR-570-3p	*IRS2*	1	1	1	1	0	1	1	6
miR-570-3p	*SLC4A4*	1	1	1	1	0	1	1	6
miR-570-3p	*VAMP4*	1	1	1	1	0	1	1	6
miR-570-3p	*PEA15*	1	1	1	1	0	1	1	6
miR-570-3p	*JRKL*	1	1	1	1	0	1	1	6
miR-570-3p	*B4GALT2*	1	1	1	1	0	1	1	6
miR-570-3p	*ABCC3*	1	1	1	1	0	1	1	6
miR-570-3p	*NOL4*	1	1	1	1	0	1	1	6
miR-570-3p	*SNX4*	1	1	1	1	0	1	1	6
miR-570-3p	*SNX3*	1	1	1	1	0	1	1	6
miR-570-3p	*ADAM19*	1	1	1	1	0	1	1	6
miR-570-3p	*CDS2*	1	1	1	1	0	1	1	6
miR-570-3p	*CD164*	1	1	1	1	0	1	1	6
miR-570-3p	*SNAP23*	1	1	1	1	0	1	1	6
miR-570-3p	*RIOK3*	1	1	1	1	0	1	1	6
miR-570-3p	*TNFRSF11A*	1	1	1	1	0	1	1	6
miR-570-3p	*TRIM24*	1	1	1	1	0	1	1	6
miR-570-3p	*DCAF5*	0	1	1	1	1	1	1	6
miR-570-3p	*CD84*	1	1	1	1	0	1	1	6
miR-570-3p	*ALKBH1*	1	1	1	1	0	1	1	6
miR-570-3p	*ASAP2*	1	1	1	1	0	1	1	6
miR-570-3p	*PER2*	1	1	1	1	0	1	1	6
miR-570-3p	*EIF2S2*	1	1	1	1	0	1	1	6
miR-570-3p	*CPNE3*	1	1	1	1	0	1	1	6
miR-570-3p	*PRPF4B*	1	1	1	1	0	1	1	6
miR-570-3p	*MPZL1*	1	1	1	1	0	1	1	6
miR-570-3p	*SEMA5A*	1	1	1	1	0	1	1	6
miR-570-3p	*MAP7*	1	1	1	1	0	1	1	6
miR-570-3p	*SLC7A6*	1	1	1	1	0	1	1	6
miR-570-3p	*CLDN8*	1	1	1	1	0	1	1	6
miR-570-3p	*MTMR4*	1	1	1	1	0	1	1	6
miR-570-3p	*SEC22C*	1	1	1	1	0	1	1	6
miR-570-3p	*RABEP1*	1	1	1	1	0	1	1	6
miR-570-3p	*OSMR*	1	1	1	1	0	1	1	6
miR-570-3p	*ZMYM6*	1	1	1	1	0	1	1	6
miR-570-3p	*XPR1*	1	1	1	1	0	1	1	6
miR-570-3p	*VAPB*	1	1	1	1	0	1	1	6

Table S1: Continued

miRNA	Gene	miRWalk	Microt4	miRanda	miRMap	RNA22	RNAhybrid	Targetscan	SUM
miR-570-3p	*MTA2*	1	1	1	1	0	1	1	6
miR-570-3p	*DLGAP2*	1	1	1	1	0	1	1	6
miR-570-3p	*DLG5*	1	1	1	1	0	1	1	6
miR-570-3p	*SRSF11*	0	1	1	1	1	1	1	6
miR-570-3p	*SOCS6*	1	1	1	1	0	1	1	6
miR-570-3p	*PPIG*	1	1	1	1	0	1	1	6
miR-570-3p	*RASAL2*	1	1	1	1	0	1	1	6
miR-570-3p	*AKAP7*	1	1	1	1	0	1	1	6
miR-570-3p	*PSMF1*	1	1	1	1	0	1	1	6
miR-570-3p	*SLC4A7*	1	1	1	1	0	1	1	6
miR-570-3p	*STXBP5L*	1	1	1	1	0	1	1	6
miR-570-3p	*IGDCC3*	1	1	1	1	0	1	1	6
miR-570-3p	*SOX13*	1	1	1	1	0	1	1	6
miR-570-3p	*PDIA4*	1	1	1	1	0	1	1	6
miR-570-3p	*AATK*	1	1	1	1	0	1	1	6
miR-570-3p	*SH3PXD2A*	0	1	1	1	1	1	1	6
miR-570-3p	*MTFR1*	1	1	1	1	0	1	1	6
miR-570-3p	*ZNF516*	1	1	1	1	0	1	1	6
miR-570-3p	*EIF5B*	1	1	1	1	0	1	1	6
miR-570-3p	*FAM53B*	1	1	1	1	0	1	1	6
miR-570-3p	*N4BP1*	1	1	1	1	0	1	1	6
miR-570-3p	*FAM131B*	1	1	1	1	0	1	1	6
miR-570-3p	*PHF16*	0	1	1	1	1	1	1	6
miR-570-3p	*KIAA0232*	1	1	1	1	0	1	1	6
miR-570-3p	*TBC1D5*	1	1	1	1	0	1	1	6
miR-570-3p	*RIMS3*	1	1	1	1	0	1	1	6
miR-570-3p	*SERTAD2*	1	1	1	1	0	1	1	6
miR-570-3p	*DAZAP2*	0	1	1	1	1	1	1	6
miR-570-3p	*KIAA0141*	1	1	1	1	0	1	1	6
miR-570-3p	*EFCAB14*	1	1	1	1	0	1	1	6
miR-570-3p	*GIT2*	0	1	1	1	1	1	1	6
miR-570-3p	*GINS1*	1	1	1	1	0	1	1	6
miR-570-3p	*TLK1*	1	1	1	1	0	1	1	6
miR-570-3p	*SMG7*	1	1	1	1	0	1	1	6
miR-570-3p	*TECPR2*	1	1	1	1	0	1	1	6
miR-570-3p	*RABGAP1L*	0	1	1	1	1	1	1	6
miR-570-3p	*HELZ*	1	1	1	1	0	1	1	6
miR-570-3p	*MAFB*	0	1	1	1	1	1	1	6
miR-570-3p	*RBM8A*	1	1	1	1	0	1	1	6
miR-570-3p	*THRAP3*	1	1	1	1	0	1	1	6

Table S1: Continued

miRNA	Gene	miRWalk	Microt4	miRanda	miRMap	RNA22	RNAhybrid	Targetscan	SUM
miR-570-3p	*HNRNPDL*	1	1	1	1	0	1	1	6
miR-570-3p	*ABI1*	1	1	1	1	0	1	1	6
miR-570-3p	*ZBTB33*	1	1	1	1	0	1	1	6
miR-570-3p	*ABCC5*	1	1	1	1	0	1	1	6
miR-570-3p	*RBM12*	1	1	1	1	0	1	1	6
miR-570-3p	*FAM13A*	1	1	1	1	0	1	1	6
miR-570-3p	*ABI2*	1	1	1	1	0	1	1	6
miR-570-3p	*WASF2*	0	1	1	1	1	1	1	6
miR-570-3p	*CNIH1*	1	1	1	1	0	1	1	6
miR-570-3p	*LHFPL2*	1	1	1	1	0	1	1	6
miR-570-3p	*AP3S2*	1	1	1	1	0	1	1	6
miR-570-3p	*KCNMB2*	1	1	1	1	0	1	1	6
miR-570-3p	*SPRY3*	1	1	1	1	0	1	1	6
miR-570-3p	*SPRY1*	1	1	1	1	0	1	1	6
miR-570-3p	*SPRY2*	1	1	1	1	0	1	1	6
miR-570-3p	*STAM2*	1	1	1	1	0	1	1	6
miR-570-3p	*Mar-06*	1	1	1	1	0	1	1	6
miR-570-3p	*RTN3*	0	1	1	1	1	1	1	6
miR-570-3p	*PCGF3*	1	1	1	1	0	1	1	6
miR-570-3p	*TUBB4B*	1	1	1	1	0	1	1	6
miR-570-3p	*CORO2B*	1	1	1	1	0	1	1	6
miR-570-3p	*ATP8A1*	1	1	1	1	0	1	1	6
miR-570-3p	*ATP8A1*	1	1	1	1	0	1	1	6
miR-570-3p	*ARIH2*	1	1	1	1	0	1	1	6
miR-570-3p	*ZBTB18*	1	1	1	1	0	1	1	6
miR-570-3p	*SLC9A6*	0	1	1	1	1	1	1	6
miR-570-3p	*SEC23A*	0	1	1	1	1	1	1	6
miR-570-3p	*NCOA2*	1	1	1	1	0	1	1	6
miR-570-3p	*DDX17*	1	1	1	1	0	1	1	6
miR-570-3p	*ARL6IP5*	1	1	1	1	0	1	1	6
miR-570-3p	*ARFGEF2*	0	1	1	1	1	1	1	6
miR-570-3p	*SMC2*	1	1	1	1	0	1	1	6
miR-570-3p	*PDLIM5*	1	1	1	1	0	1	1	6
miR-570-3p	*HEXIM1*	1	1	1	1	0	1	1	6
miR-570-3p	*STAMBP*	1	1	1	1	0	1	1	6
miR-570-3p	*CELF1*	1	1	1	1	0	1	1	6
miR-570-3p	*CELF2*	1	1	1	1	0	1	1	6
miR-570-3p	*NFAT5*	0	1	1	1	1	1	1	6
miR-570-3p	*HBS1L*	1	1	1	1	0	1	1	6
miR-570-3p	*FRS2*	1	1	1	1	0	1	1	6

Table S1: Continued

miRNA	Gene	miRWalk	Microt4	miRanda	miRMap	RNA22	RNAhybrid	Targetscan	SUM
miR-570-3p	*SUB1*	1	1	1	1	0	1	1	6
miR-570-3p	*SPIN1*	1	1	1	1	0	1	1	6
miR-570-3p	*GCN1L1*	1	1	1	1	0	1	1	6
miR-570-3p	*KIF2C*	1	1	1	1	0	1	1	6
miR-570-3p	*NUDT21*	0	1	1	1	1	1	1	6
miR-570-3p	*ABHD2*	1	1	1	1	0	1	1	6
miR-570-3p	*RAPGEF4*	1	1	1	1	0	1	1	6
miR-570-3p	*ADAMTS5*	1	1	1	1	0	1	1	6
miR-570-3p	*HNRNPUL1*	1	1	1	1	0	1	1	6
miR-570-3p	*PTPRT*	1	1	1	1	0	1	1	6
miR-570-3p	*ZWINT*	1	1	1	1	0	1	1	6
miR-570-3p	*AP4S1*	1	1	1	1	0	1	1	6
miR-570-3p	*MAP4K5*	0	1	1	1	1	1	1	6
miR-570-3p	*AKAP13*	0	1	1	1	1	1	1	6
miR-570-3p	*AKAP11*	1	1	1	1	0	1	1	6
miR-570-3p	*SEC63*	1	1	1	1	0	1	1	6
miR-570-3p	*KLF12*	1	1	1	1	0	1	1	6
miR-570-3p	*KLF8*	1	1	1	1	0	1	1	6
miR-570-3p	*MGAT4A*	1	1	1	1	0	1	1	6
miR-570-3p	*DDX42*	1	1	1	1	0	1	1	6
miR-570-3p	*RNF13*	1	1	1	1	0	1	1	6
miR-570-3p	*RAB11FIP2*	1	1	1	1	0	1	1	6
miR-570-3p	*VASH1*	1	1	1	1	0	1	1	6
miR-570-3p	*ADNP2*	1	1	1	1	0	1	1	6
miR-570-3p	*CHSY1*	1	1	1	1	0	1	1	6
miR-570-3p	*NLGN1*	1	1	1	1	0	1	1	6
miR-570-3p	*ENPP4*	0	1	1	1	1	1	1	6
miR-570-3p	*INPP5F*	1	1	1	1	0	1	1	6
miR-570-3p	*FOXJ3*	1	1	1	1	0	1	1	6
miR-570-3p	*ZNF365*	0	1	1	1	1	1	1	6
miR-570-3p	*MAPRE1*	1	1	1	1	0	1	1	6
miR-570-3p	*ATF6*	1	1	1	1	0	1	1	6
miR-570-3p	*SEPHS1*	1	1	1	1	0	1	1	6
miR-570-3p	*CCT5*	1	1	1	1	0	1	1	6
miR-570-3p	*RIMS1*	1	1	1	1	0	1	1	6
miR-570-3p	*WDFY3*	1	1	1	1	0	1	1	6
miR-570-3p	*TMCC1*	1	1	1	1	0	1	1	6
miR-570-3p	*KDM4B*	1	1	1	1	0	1	1	6
miR-570-3p	*SAMD4A*	1	1	1	1	0	1	1	6
miR-570-3p	*PHLPP2*	1	1	1	1	0	1	1	6

Table S1: Continued

miRNA	Gene	miRWalk	Microt4	miRanda	miRMap	RNA22	RNAhybrid	Targetscan	SUM
miR-570-3p	*WDTC1*	1	1	1	1	0	1	1	6
miR-570-3p	*MYT1L*	1	1	1	0	1	1	1	6
miR-570-3p	*SWAP70*	1	1	1	1	0	1	1	6
miR-570-3p	*AVL9*	1	1	1	1	0	1	1	6
miR-570-3p	*TRIM35*	1	1	1	1	0	1	1	6
miR-570-3p	*CDK19*	1	1	1	1	0	1	1	6
miR-570-3p	*TNRC6B*	1	1	1	1	0	1	1	6
miR-570-3p	*NFASC*	1	1	1	1	0	1	1	6
miR-570-3p	*HIC2*	1	1	1	1	0	1	1	6
miR-570-3p	*ZZEF1*	1	1	1	1	0	1	1	6
miR-570-3p	*DCUN1D4*	1	1	1	1	0	1	1	6
miR-570-3p	*SYT11*	1	1	1	1	0	1	1	6
miR-570-3p	*ZC3H4*	0	1	1	1	1	1	1	6
miR-570-3p	*FBXO28*	1	1	1	1	0	1	1	6
miR-570-3p	*DTX4*	1	1	1	1	0	1	1	6
miR-570-3p	*ARHGEF9*	1	1	1	1	0	1	1	6
miR-570-3p	*PLCB1*	1	1	1	1	0	1	1	6
miR-570-3p	*ANKRD12*	1	1	1	1	0	1	1	6
miR-570-3p	*MGA*	1	1	1	1	0	1	1	6
miR-570-3p	*CUX2*	1	1	1	1	0	1	1	6
miR-570-3p	*USP22*	1	1	1	1	0	1	1	6
miR-570-3p	*NEDD4L*	1	1	1	1	0	1	1	6
miR-570-3p	*KIAA0895*	0	1	1	1	1	1	1	6
miR-570-3p	*MED13L*	1	1	1	1	0	1	1	6
miR-570-3p	*EXOSC2*	1	1	1	1	0	1	1	6
miR-570-3p	*DICER1*	1	1	1	1	0	1	1	6
miR-570-3p	*SIRT1*	1	1	1	1	0	1	1	6
miR-570-3p	*SLC44A1*	1	1	1	1	0	1	1	6
miR-570-3p	*CBX5*	0	1	1	1	1	1	1	6
miR-570-3p	*TRAM1*	1	1	1	1	0	1	1	6
miR-570-3p	*SEZ6L*	0	1	1	1	1	1	1	6
miR-570-3p	*DDAH1*	1	1	1	1	0	1	1	6
miR-570-3p	*PRND*	1	1	1	1	0	1	1	6
miR-570-3p	*SSBP3*	1	1	1	1	0	1	1	6
miR-570-3p	*STX12*	1	1	1	1	0	1	1	6
miR-570-3p	*SH3BP4*	1	1	1	1	0	1	1	6
miR-570-3p	*MTCH2*	0	1	1	1	1	1	1	6
miR-570-3p	*METTL7A*	1	1	1	1	0	1	1	6
miR-570-3p	*MOB4*	0	1	1	1	1	1	1	6
miR-570-3p	*RNF19A*	0	1	1	1	1	1	1	6

Table S1: Continued

miRNA	Gene	miRWalk	Microt4	miRanda	miRMap	RNA22	RNAhybrid	Targetscan	SUM
miR-570-3p	*THUMPD3*	0	1	1	1	1	1	1	6
miR-570-3p	*C20orf194*	1	1	1	1	0	1	1	6
miR-570-3p	*GPR124*	1	1	1	1	0	1	1	6
miR-570-3p	*NUDT13*	1	1	1	1	0	1	1	6
miR-570-3p	*C2CD2*	1	1	1	1	0	1	1	6
miR-570-3p	*NGDN*	1	1	1	1	0	1	1	6
miR-570-3p	*L3MBTL1*	1	1	1	1	0	1	1	6
miR-570-3p	*LRP10*	1	1	1	1	0	1	1	6
miR-570-3p	*SS18L1*	0	1	1	1	1	1	1	6
miR-570-3p	*FAM169A*	1	1	1	1	0	1	1	6
miR-570-3p	*PPP1R16B*	1	1	1	1	0	1	1	6
miR-570-3p	*AUTS2*	1	1	1	1	0	1	1	6
miR-570-3p	*RAB11FIP5*	1	1	1	1	0	1	1	6
miR-570-3p	*RAI14*	0	1	1	1	1	1	1	6
miR-570-3p	*LSM14A*	1	1	1	1	0	1	1	6
miR-570-3p	*SZRD1*	0	1	1	1	1	1	1	6
miR-570-3p	*FBXL4*	1	1	1	1	0	1	1	6
miR-570-3p	*BLOC1S6*	1	1	1	1	0	1	1	6
miR-570-3p	*FBXO3*	1	1	1	1	0	1	1	6
miR-570-3p	*GREM1*	1	1	1	1	0	1	1	6
miR-570-3p	*ZBTB11*	1	1	1	1	0	1	1	6
miR-570-3p	*KLHL20*	1	1	1	1	0	1	1	6
miR-570-3p	*PCDH17*	1	1	1	1	0	1	1	6
miR-570-3p	*PCDH11X*	0	1	1	1	1	1	1	6
miR-570-3p	*HIPK2*	1	1	1	1	0	1	1	6
miR-570-3p	*CCDC113*	1	1	1	1	0	1	1	6
miR-570-3p	*TMOD3*	1	1	1	1	0	1	1	6
miR-570-3p	*TFCP2L1*	1	1	1	1	0	1	1	6
miR-570-3p	*SENP1*	1	1	1	1	0	1	1	6
miR-570-3p	*CNOT7*	1	1	1	1	0	1	1	6
miR-570-3p	*SNX12*	0	1	1	1	1	1	1	6
miR-570-3p	*HUNK*	1	1	1	1	0	1	1	6
miR-570-3p	*ST6GALNAC6*	1	1	1	1	0	1	1	6
miR-570-3p	*ZBTB21*	0	1	1	1	1	1	1	6
miR-570-3p	*MYEF2*	1	1	1	1	0	1	1	6
miR-570-3p	*CDON*	1	1	1	1	0	1	1	6
miR-570-3p	*TMED5*	1	1	1	1	0	1	1	6
miR-570-3p	*TMED7*	1	1	1	1	0	1	1	6
miR-570-3p	*ST8SIA3*	1	1	1	1	0	1	1	6
miR-570-3p	*SEPSECS*	1	1	1	1	0	1	1	6

Table S1: Continued

miRNA	Gene	miRWalk	Microt4	miRanda	miRMap	RNA22	RNAhybrid	Targetscan	SUM
miR-570-3p	*SH3GLB1*	1	1	1	1	0	1	1	6
miR-570-3p	*SAR1B*	0	1	1	1	1	1	1	6
miR-570-3p	*PHF20*	1	1	1	1	0	1	1	6
miR-570-3p	*CRIM1*	1	1	1	1	0	1	1	6
miR-570-3p	*NIP7*	1	1	1	1	0	1	1	6
miR-570-3p	*COMMD10*	1	1	1	1	0	1	1	6
miR-570-3p	*FAM8A1*	1	1	1	1	0	1	1	6
miR-570-3p	*CTDSPL2*	1	1	1	1	0	1	1	6
miR-570-3p	*VPS54*	1	1	1	1	0	1	1	6
miR-570-3p	*RAB14*	1	1	1	1	0	1	1	6
miR-570-3p	*PEX5L*	1	1	1	1	0	1	1	6
miR-570-3p	*TRIM33*	1	1	1	1	0	1	1	6
miR-570-3p	*KLF13*	1	1	1	1	0	1	1	6
miR-570-3p	*ATP8A2*	1	1	1	1	0	1	1	6
miR-570-3p	*TM6SF1*	1	1	1	1	0	1	1	6
miR-570-3p	*LRP1B*	1	1	1	1	0	1	1	6
miR-570-3p	*CPSF2*	0	1	1	1	1	1	1	6
miR-570-3p	*BRWD1*	0	1	1	1	1	1	1	6
miR-570-3p	*C21orf91*	0	1	1	1	1	1	1	6
miR-570-3p	*ATAD2B*	1	1	1	1	0	1	1	6
miR-570-3p	*ANKIB1*	1	1	1	1	0	1	1	6
miR-570-3p	*SPATA6*	1	1	1	1	0	1	1	6
miR-570-3p	*MAP10*	1	1	1	1	0	1	1	6
miR-570-3p	*TMEM106B*	1	1	1	0	1	1	1	6
miR-570-3p	*RSBN1*	1	1	1	1	0	1	1	6
miR-570-3p	*CRLS1*	0	1	1	1	1	1	1	6
miR-570-3p	*Mar-05*	1	1	1	1	0	1	1	6
miR-570-3p	*OTUD4*	1	1	1	1	0	1	1	6
miR-570-3p	*IL17RD*	1	1	1	1	0	1	1	6
miR-570-3p	*ZRANB1*	1	1	1	1	0	1	1	6
miR-570-3p	*DNAJB12*	0	1	1	1	1	1	1	6
miR-570-3p	*TET2*	1	1	1	1	0	1	1	6
miR-570-3p	*BNC2*	1	1	1	1	0	1	1	6
miR-570-3p	*BIVM*	1	1	1	1	0	1	1	6
miR-570-3p	*PIGG*	1	1	1	1	0	1	1	6
miR-570-3p	*DPP8*	1	1	1	1	0	1	1	6
miR-570-3p	*LPPR1*	1	1	1	1	0	1	1	6
miR-570-3p	*INO80D*	1	1	1	1	0	1	1	6
miR-570-3p	*CMTM6*	1	1	1	1	0	1	1	6
miR-570-3p	*IMPAD1*	1	1	1	1	0	1	1	6

Table S1: Continued

miRNA	Gene	miRWalk	Microt4	miRanda	miRMap	RNA22	RNAhybrid	Targetscan	SUM
miR-570-3p	*PARP16*	1	1	1	1	0	1	1	6
miR-570-3p	*SLC35F6*	1	1	1	1	0	1	1	6
miR-570-3p	*PLEKHB2*	1	1	1	1	0	1	1	6
miR-570-3p	*PDPR*	1	1	1	1	0	1	1	6
miR-570-3p	*DET1*	1	1	1	1	0	1	1	6
miR-570-3p	*C10orf118*	1	1	1	1	0	1	1	6
miR-570-3p	*PRPF38B*	1	1	1	1	0	1	1	6
miR-570-3p	*LARP1B*	0	1	1	1	1	1	1	6
miR-570-3p	*MSL2*	0	1	1	1	1	1	1	6
miR-570-3p	*SMG8*	0	1	1	1	1	1	1	6
miR-570-3p	*SBNO1*	1	1	1	1	0	1	1	6
miR-570-3p	*SMU1*	0	1	1	1	1	1	1	6
miR-570-3p	*VPS53*	1	1	1	1	0	1	1	6
miR-570-3p	*ZNF654*	1	1	1	1	0	1	1	6
miR-570-3p	*LIN7C*	1	1	1	1	0	1	1	6
miR-570-3p	*YOD1*	1	1	1	1	0	1	1	6
miR-570-3p	*ETNK1*	1	1	1	1	0	1	1	6
miR-570-3p	*ELMOD1*	1	1	1	1	0	1	1	6
miR-570-3p	*SOX6*	1	1	1	1	0	1	1	6
miR-570-3p	*FAM46A*	1	1	1	1	0	1	1	6
miR-570-3p	*ZNF407*	0	1	1	1	1	1	1	6
miR-570-3p	*G2E3*	1	1	1	1	0	1	1	6
miR-570-3p	*SLC30A6*	1	1	1	1	0	1	1	6
miR-570-3p	*WDR11*	1	1	1	1	0	1	1	6
miR-570-3p	*Sep-11*	1	1	1	1	0	1	1	6
miR-570-3p	*TMEM30A*	1	1	1	1	0	1	1	6
miR-570-3p	*MCTP2*	1	1	1	1	0	1	1	6
miR-570-3p	*FGD6*	1	1	1	1	0	1	1	6
miR-570-3p	*TRERF1*	1	1	1	1	0	1	1	6
miR-570-3p	*UBAP2*	1	1	1	1	0	1	1	6
miR-570-3p	*ASH1L*	1	1	1	1	0	1	1	6
miR-570-3p	*ZNF395*	1	1	1	1	0	1	1	6
miR-570-3p	*CTTNBP2NL*	1	1	1	1	0	1	1	6
miR-570-3p	*PCDHA6*	1	1	1	1	0	1	1	6
miR-570-3p	*LMOD3*	1	1	1	1	0	1	1	6
miR-570-3p	*CLDND1*	1	1	1	1	0	1	1	6
miR-570-3p	*JPH1*	1	1	1	1	0	1	1	6
miR-570-3p	*SPIRE1*	1	1	1	1	0	1	1	6
miR-570-3p	*CA10*	1	1	1	1	0	1	1	6
miR-570-3p	*PMEPA1*	0	1	1	1	1	1	1	6

Table S1: Continued

miRNA	Gene	miRWalk	Microt4	miRanda	miRMap	RNA22	RNAhybrid	Targetscan	SUM
miR-570-3p	*LHX9*	1	1	1	1	0	1	1	6
miR-570-3p	*STOX2*	1	1	1	1	0	1	1	6
miR-570-3p	*BBX*	1	1	1	1	0	1	1	6
miR-570-3p	*CDC42SE2*	1	1	1	1	0	1	1	6
miR-570-3p	*TWSG1*	1	1	1	1	0	1	1	6
miR-570-3p	*GOPC*	1	1	1	1	0	1	1	6
miR-570-3p	*SLC39A10*	1	1	1	1	0	1	1	6
miR-570-3p	*KIAA1244*	1	1	0	1	1	1	1	6
miR-570-3p	*ZNF286A*	1	1	1	1	0	1	1	6
miR-570-3p	*TMCC3*	1	1	1	1	0	1	1	6
miR-570-3p	*GATAD2B*	1	1	1	1	0	1	1	6
miR-570-3p	*PPM1H*	1	1	1	1	0	1	1	6
miR-570-3p	*ERMN*	1	1	1	1	0	1	1	6
miR-570-3p	*GRAMD1B*	1	1	1	1	0	1	1	6
miR-570-3p	*NLN*	1	1	1	1	0	1	1	6
miR-570-3p	*RIMKLB*	1	1	1	1	0	1	1	6
miR-570-3p	*MKL2*	1	1	1	1	0	1	1	6
miR-570-3p	*MTA3*	1	1	1	1	0	1	1	6
miR-570-3p	*INTS2*	1	1	1	1	0	1	1	6
miR-570-3p	*NUFIP2*	1	1	1	1	0	1	1	6
miR-570-3p	*MIB1*	1	1	1	1	0	1	1	6
miR-570-3p	*SEMA6A*	0	1	1	1	1	1	1	6
miR-570-3p	*KLHL14*	0	1	1	1	1	1	1	6
miR-570-3p	*TMEM181*	1	1	1	1	0	1	1	6
miR-570-3p	*KIAA1430*	1	1	1	1	0	1	1	6
miR-570-3p	*WDFY1*	1	1	1	1	0	1	1	6
miR-570-3p	*FNIP2*	1	1	1	1	0	1	1	6
miR-570-3p	*RANBP10*	1	1	1	1	0	1	1	6
miR-570-3p	*ISLR2*	1	1	1	1	0	1	1	6
miR-570-3p	*SHROOM3*	1	1	1	1	0	1	1	6
miR-570-3p	*DPP10*	0	1	1	1	1	1	1	6
miR-570-3p	*FBRSL1*	1	1	1	1	0	1	1	6
miR-570-3p	*KIAA1549*	1	1	1	1	0	1	1	6
miR-570-3p	*GPAM*	1	1	1	1	0	1	1	6
miR-570-3p	*KIAA1598*	1	1	1	1	0	1	1	6
miR-570-3p	*FAM160B1*	1	1	1	1	0	1	1	6
miR-570-3p	*SLC7A14*	1	1	1	1	0	1	1	6
miR-570-3p	*SFMBT2*	1	1	1	1	0	1	1	6
miR-570-3p	*GPR107*	1	1	1	1	0	1	1	6
miR-570-3p	*EPG5*	1	1	1	1	0	1	1	6

Table S1: Continued

miRNA	Gene	miRWalk	Microt4	miRanda	miRMap	RNA22	RNAhybrid	Targetscan	SUM
miR-570-3p	*TP53INP2*	1	1	1	1	0	1	1	6
miR-570-3p	*FAM60A*	1	1	1	1	0	1	1	6
miR-570-3p	*SNX6*	1	1	1	1	0	1	1	6
miR-570-3p	*TGIF2*	1	1	1	1	0	1	1	6
miR-570-3p	*BACH2*	1	1	1	1	0	1	1	6
miR-570-3p	*BCORL1*	1	1	1	1	0	1	1	6
miR-570-3p	*PRDM15*	0	1	1	1	1	1	1	6
miR-570-3p	*NPAS3*	1	1	1	1	0	1	1	6
miR-570-3p	*C10orf54*	1	1	1	1	0	1	1	6
miR-570-3p	*XYLT1*	1	1	1	1	0	1	1	6
miR-570-3p	*NECAB1*	1	1	1	1	0	1	1	6
miR-570-3p	*PAPD5*	1	1	1	1	0	1	1	6
miR-570-3p	*ZMAT3*	1	1	1	1	0	1	1	6
miR-570-3p	*COPS7B*	1	1	1	1	0	1	1	6
miR-570-3p	*ACBD3*	1	1	1	1	0	1	1	6
miR-570-3p	*RMND5A*	1	1	1	1	0	1	1	6
miR-570-3p	*YTHDC2*	1	1	1	1	0	1	1	6
miR-570-3p	*USP46*	1	1	1	1	0	1	1	6
miR-570-3p	*RFX7*	1	1	1	1	0	1	1	6
miR-570-3p	*RANBP17*	1	1	1	1	0	1	1	6
miR-570-3p	*CASD1*	1	1	1	1	0	1	1	6
miR-570-3p	*REEP1*	1	1	1	1	0	1	1	6
miR-570-3p	*WNK3*	1	1	1	1	0	1	1	6
miR-570-3p	*ZBTB10*	1	1	1	1	0	1	1	6
miR-570-3p	*MTMR9*	1	1	1	1	0	1	1	6
miR-570-3p	*BOLL*	1	1	1	1	0	1	1	6
miR-570-3p	*TMEM43*	1	1	1	1	0	1	1	6
miR-570-3p	*RNF219*	1	1	1	1	0	1	1	6
miR-570-3p	*RIC3*	1	1	1	1	0	1	1	6
miR-570-3p	*ARHGAP10*	1	1	1	1	0	1	1	6
miR-570-3p	*ZMAT4*	1	1	1	1	0	1	1	6
miR-570-3p	*ZYG11B*	1	1	1	1	0	1	1	6
miR-570-3p	*TBL1XR1*	1	1	1	1	0	1	1	6
miR-570-3p	*SNIP1*	1	1	1	1	0	1	1	6
miR-570-3p	*MOB3B*	1	1	1	1	0	1	1	6
miR-570-3p	*THSD4*	1	1	1	1	0	1	1	6
miR-570-3p	*MCMBP*	1	1	1	1	0	1	1	6
miR-570-3p	*CYBRD1*	1	1	1	1	0	1	1	6
miR-570-3p	*ERMP1*	1	1	1	1	0	1	1	6
miR-570-3p	*C10orf88*	1	1	0	1	1	1	1	6

Table S1: Continued

miRNA	Gene	miRWalk	Microt4	miRanda	miRMap	RNA22	RNAhybrid	Targetscan	SUM
miR-570-3p	*PHC3*	1	1	1	1	0	1	1	6
miR-570-3p	*WWC2*	1	1	1	1	0	1	1	6
miR-570-3p	*TRPM3*	1	1	1	1	0	1	1	6
miR-570-3p	*PGAP1*	1	1	1	1	0	1	1	6
miR-570-3p	*CCDC176*	1	1	1	1	0	1	1	6
miR-570-3p	*CCDC170*	1	1	1	1	0	1	1	6
miR-570-3p	*SIKE1*	1	1	1	1	0	1	1	6
miR-570-3p	*WDR26*	1	1	1	1	0	1	1	6
miR-570-3p	*EPC1*	1	1	1	1	0	1	1	6
miR-570-3p	*KCNIP4*	1	1	1	1	0	1	1	6
miR-570-3p	*CYB5B*	1	1	1	1	0	1	1	6
miR-570-3p	*DDHD1*	1	1	1	1	0	1	1	6
miR-570-3p	*ZFP91*	0	1	1	1	1	1	1	6
miR-570-3p	*JHDM1D*	1	1	1	1	0	1	1	6
miR-570-3p	*FAM49A*	1	1	1	1	0	1	1	6
miR-570-3p	*FAM117A*	1	1	1	1	0	1	1	6
miR-570-3p	*SNX27*	1	1	1	1	0	1	1	6
miR-570-3p	*ANP32E*	1	1	1	1	0	1	1	6
miR-570-3p	*MAP1LC3B*	1	1	1	1	0	1	1	6
miR-570-3p	*VANGL1*	1	1	1	1	0	1	1	6
miR-570-3p	*SEH1L*	1	1	1	1	0	1	1	6
miR-570-3p	*PCDH11Y*	0	1	1	1	1	1	1	6
miR-570-3p	*RAB33B*	1	1	1	1	0	1	1	6
miR-570-3p	*FAM167A*	1	1	1	1	0	1	1	6
miR-570-3p	*CALN1*	1	1	1	1	0	1	1	6
miR-570-3p	*SH3BGRL2*	0	1	1	1	1	1	1	6
miR-570-3p	*SETDB2*	1	1	1	1	0	1	1	6
miR-570-3p	*FAM172A*	1	1	1	1	0	1	1	6
miR-570-3p	*B3GNT5*	1	1	1	1	0	1	1	6
miR-570-3p	*MAGT1*	1	1	1	1	0	1	1	6
miR-570-3p	*SLC10A7*	1	1	1	1	0	1	1	6
miR-570-3p	*LRRIQ1*	1	1	1	1	0	1	1	6
miR-570-3p	*UTP15*	1	1	1	1	0	1	1	6
miR-570-3p	*RNASEH2C*	0	1	1	1	1	1	1	6
miR-570-3p	*ARID5B*	0	1	1	1	1	1	1	6
miR-570-3p	*HSDL2*	1	1	1	1	0	1	1	6
miR-570-3p	*PHF6*	1	1	1	1	0	1	1	6
miR-570-3p	*SARNP*	1	1	1	1	0	1	1	6
miR-570-3p	*RAB11FIP4*	1	1	1	1	0	1	1	6
miR-570-3p	*PHYHIPL*	1	1	1	1	0	1	1	6

Table S1: Continued

miRNA	Gene	miRWalk	Microt4	miRanda	miRMap	RNA22	RNAhybrid	Targetscan	SUM
miR-570-3p	*LCOR*	0	1	1	1	1	1	1	6
miR-570-3p	*FAXC*	1	1	1	1	0	1	1	6
miR-570-3p	*PARD6B*	1	1	1	1	0	1	1	6
miR-570-3p	*RNASE7*	1	1	1	1	0	1	1	6
miR-570-3p	*FAM136A*	1	1	1	1	0	1	1	6
miR-570-3p	*ZNRF1*	1	1	1	1	0	1	1	6
miR-570-3p	*KIAA1671*	1	1	1	1	0	1	1	6
miR-570-3p	*KIAA1737*	1	1	1	1	0	1	1	6
miR-570-3p	*ZC3H12C*	1	1	1	1	0	1	1	6
miR-570-3p	*SSH2*	1	1	1	1	0	1	1	6
miR-570-3p	*SSH2*	1	1	1	1	0	1	1	6
miR-570-3p	*EPT1*	1	1	1	1	0	1	1	6
miR-570-3p	*NAV2*	1	1	1	1	0	1	1	6
miR-570-3p	*MIDN*	1	1	1	1	0	1	1	6
miR-570-3p	*CCDC120*	1	1	1	1	0	1	1	6
miR-570-3p	*KCNH7*	1	1	1	1	0	1	1	6
miR-570-3p	*HS6ST2*	1	1	1	1	0	1	1	6
miR-570-3p	*FAM105B*	1	1	1	1	0	1	1	6
miR-570-3p	*STARD13*	0	1	1	1	1	1	1	6
miR-570-3p	*ZNF697*	1	1	1	1	0	1	1	6
miR-570-3p	*SESTD1*	1	1	1	1	0	1	1	6
miR-570-3p	*PLXNA4*	1	1	1	1	0	1	1	6
miR-570-3p	*ELMSAN1*	1	1	1	1	0	1	1	6
miR-570-3p	*CABLES1*	1	1	1	1	0	1	1	6
miR-570-3p	*HNRNPLL*	1	1	1	1	0	1	1	6
miR-570-3p	*MYOCD*	1	1	1	1	0	1	1	6
miR-570-3p	*DTD2*	1	1	1	1	0	1	1	6
miR-570-3p	*FMNL2*	1	1	1	1	0	1	1	6
miR-570-3p	*ELFN2*	1	1	1	1	0	1	1	6
miR-570-3p	*ZNF618*	1	1	1	1	0	1	1	6
miR-570-3p	*SLC26A7*	1	1	1	1	0	1	1	6
miR-570-3p	*KCTD12*	1	1	1	1	0	1	1	6
miR-570-3p	*FCRL3*	1	1	1	1	0	1	1	6
miR-570-3p	*CYYR1*	0	1	1	1	1	1	1	6
miR-570-3p	*C8orf34*	1	1	1	1	0	1	1	6
miR-570-3p	*RAB3IP*	1	1	1	1	0	1	1	6
miR-570-3p	*ANTXR2*	1	1	1	1	0	1	1	6
miR-570-3p	*C10orf90*	1	1	1	1	0	1	1	6
miR-570-3p	*SOCS4*	0	1	1	1	1	1	1	6
miR-570-3p	*TC2N*	1	1	1	1	0	1	1	6

Table S1: Continued

miRNA	Gene	miRWalk	Microt4	miRanda	miRMap	RNA22	RNAhybrid	Targetscan	SUM
miR-570-3p	*NLRP5*	1	1	1	1	0	1	1	6
miR-570-3p	*SYT2*	1	1	1	1	0	1	1	6
miR-570-3p	*UHMK1*	1	1	1	1	0	1	1	6
miR-570-3p	*LIX1L*	1	1	1	1	0	1	1	6
miR-570-3p	*TBC1D20*	1	1	1	1	0	1	1	6
miR-570-3p	*DUSP15*	1	1	1	1	0	1	1	6
miR-570-3p	*FAM168B*	1	1	0	1	1	1	1	6
miR-570-3p	*LYPD6*	1	1	1	1	0	1	1	6
miR-570-3p	*KCNH8*	1	1	1	1	0	1	1	6
miR-570-3p	*PPARGC1B*	1	1	1	1	0	1	1	6
miR-570-3p	*STARD4*	1	1	1	1	0	1	1	6
miR-570-3p	*WDR36*	1	1	1	1	0	1	1	6
miR-570-3p	*C5orf24*	1	1	1	1	0	1	1	6
miR-570-3p	*CD109*	1	1	1	1	0	1	1	6
miR-570-3p	*MTPN*	1	1	1	1	0	1	1	6
miR-570-3p	*UBXN2B*	1	1	1	1	0	1	1	6
miR-570-3p	*NACC2*	1	1	1	1	0	1	1	6
miR-570-3p	*SLITRK4*	1	1	1	1	0	1	1	6
miR-570-3p	*SOGA1*	0	1	1	1	1	1	1	6
miR-570-3p	*ZNF280B*	1	1	1	1	0	1	1	6
miR-570-3p	*PABPC5*	1	1	1	1	0	1	1	6
miR-570-3p	*CACUL1*	0	1	1	1	1	1	1	6
miR-570-3p	*SESN3*	1	1	1	1	0	1	1	6
miR-570-3p	*E2F7*	1	1	1	1	0	1	1	6
miR-570-3p	*CMTM4*	1	1	1	1	0	1	1	6
miR-570-3p	*RUNDC1*	1	1	1	1	0	1	1	6
miR-570-3p	*C18orf25*	1	1	1	1	0	1	1	6
miR-570-3p	*CCBE1*	1	1	1	1	0	1	1	6
miR-570-3p	*SYT6*	1	1	1	1	0	1	1	6
miR-570-3p	*PHF13*	1	1	1	1	0	1	1	6
miR-570-3p	*SLC30A7*	1	1	1	1	0	1	1	6
miR-570-3p	*PDIK1L*	1	1	1	1	0	1	1	6
miR-570-3p	*CCDC117*	1	1	1	1	0	1	1	6
miR-570-3p	*ARL6IP6*	1	1	1	1	0	1	1	6
miR-570-3p	*CCNYL1*	1	1	1	1	0	1	1	6
miR-570-3p	*PPP1R1C*	1	1	1	1	0	1	1	6
miR-570-3p	*TTC14*	1	1	1	1	0	1	1	6
miR-570-3p	*CCDC50*	1	1	1	1	0	1	1	6
miR-570-3p	*CNTN4*	1	1	1	1	0	1	1	6
miR-570-3p	*NFXL1*	0	1	1	1	1	1	1	6

Table S1: Continued

miRNA	Gene	miRWalk	Microt4	miRanda	miRMap	RNA22	RNAhybrid	Targetscan	SUM
miR-570-3p	*CREBRF*	1	1	1	1	0	1	1	6
miR-570-3p	*RNF217*	1	1	1	1	0	1	1	6
miR-570-3p	*AMOT*	1	1	1	1	0	1	1	6
miR-570-3p	*TMTC3*	1	1	1	1	0	1	1	6
miR-570-3p	*GPR180*	1	1	1	1	0	1	1	6
miR-570-3p	*ZNF709*	1	1	1	1	0	1	1	6
miR-570-3p	*DENND1B*	1	1	1	1	0	1	1	6
miR-570-3p	*LONRF2*	1	1	1	1	0	1	1	6
miR-570-3p	*PRICKLE2*	1	1	1	1	0	1	1	6
miR-570-3p	*SGMS2*	1	1	1	1	0	1	1	6
miR-570-3p	*TXLNB*	1	1	1	1	0	1	1	6
miR-570-3p	*THAP5*	0	1	1	1	1	1	1	6
miR-570-3p	*ZNF800*	1	1	1	1	0	1	1	6
miR-570-3p	*TMEM64*	0	1	1	1	1	1	1	6
miR-570-3p	*OLFML2A*	1	1	1	1	0	1	1	6
miR-570-3p	*GLIS3*	1	1	1	1	0	1	1	6
miR-570-3p	*VSTM4*	1	1	1	1	0	1	1	6
miR-570-3p	*LCTL*	1	1	1	1	0	1	1	6
miR-570-3p	*CC2D1B*	0	1	1	1	1	1	1	6
miR-570-3p	*TXLNA*	1	1	1	1	0	1	1	6
miR-570-3p	*STK32A*	0	1	1	1	1	1	1	6
miR-570-3p	*C9orf72*	1	1	1	1	0	1	1	6
miR-570-3p	*ANKS6*	0	1	1	1	1	1	1	6
miR-570-3p	*ZNF449*	1	1	1	1	0	1	1	6
miR-570-3p	*VMA21*	1	1	1	1	0	1	1	6
miR-570-3p	*HIPK1*	0	1	1	1	1	1	1	6
miR-570-3p	*HIPK1*	0	1	1	1	1	1	1	6
miR-570-3p	*C2orf69*	1	1	1	1	0	1	1	6
miR-570-3p	*ZNF25*	1	1	1	1	0	1	1	6
miR-570-3p	*RTKN2*	1	1	1	1	0	1	1	6
miR-570-3p	*DOK6*	1	1	1	1	0	1	1	6
miR-570-3p	*HNRNPA3*	1	1	1	1	0	1	1	6
miR-570-3p	*ARL5B*	1	1	1	1	0	1	1	6
miR-570-3p	*HNRNPUL2*	1	1	1	1	0	1	1	6
miR-570-3p	*RBM24*	1	1	1	1	0	1	1	6
miR-570-3p	*SMIM13*	1	1	1	1	0	1	1	6
miR-570-3p	*FOXK1*	1	1	1	1	0	1	1	6
miR-570-3p	*HS3ST5*	1	1	1	1	0	1	1	6
miR-570-3p	*SLC35F1*	1	1	1	1	0	1	1	6
miR-570-3p	*ZSCAN23*	1	1	1	1	0	1	1	6

Table S1: Continued

miRNA	Gene	miRWalk	Microt4	miRanda	miRMap	RNA22	RNAhybrid	Targetscan	SUM
miR-570-3p	*ZNRF2*	1	1	1	1	0	1	1	6
miR-570-3p	*CNOT6L*	1	1	1	1	0	1	1	6
miR-570-3p	*SLC25A30*	0	1	1	1	1	1	1	6
miR-570-3p	*LCLAT1*	1	1	1	1	0	1	1	6
miR-570-3p	*CADM2*	1	1	1	1	0	1	1	6
miR-570-3p	*MMS22L*	1	1	1	1	0	1	1	6
miR-570-3p	*CERS6*	1	1	1	1	0	1	1	6
miR-570-3p	*UBN2*	1	1	1	1	0	1	1	6
miR-570-3p	*MCOLN2*	1	1	1	1	0	1	1	6
miR-570-3p	*ELMOD2*	0	1	1	1	1	1	1	6
miR-570-3p	*SYT14*	1	1	1	1	0	1	1	6
miR-570-3p	*LYSMD2*	1	1	1	1	0	1	1	6
miR-570-3p	*MAP7D2*	0	1	1	1	1	1	1	6
miR-570-3p	*RNF214*	1	1	1	1	0	1	1	6
miR-570-3p	*HSPA12A*	0	1	1	1	1	1	1	6
miR-570-3p	*RBM20*	1	1	1	1	0	1	1	6
miR-570-3p	*MKX*	1	1	1	1	0	1	1	6
miR-570-3p	*PGM2L1*	1	1	1	1	0	1	1	6
miR-570-3p	*OLFML1*	1	1	1	1	0	1	1	6
miR-570-3p	*RASSF3*	1	1	1	1	0	1	1	6
miR-570-3p	*KSR2*	1	1	1	1	0	1	1	6
miR-570-3p	*PRTG*	1	1	1	1	0	1	1	6
miR-570-3p	*UNC80*	1	1	1	1	0	1	1	6
miR-570-3p	*C5orf51*	0	1	1	1	1	1	1	6
miR-570-3p	*RGMB*	1	1	1	1	0	1	1	6
miR-570-3p	*TRIQK*	1	1	1	1	0	1	1	6
miR-570-3p	*DPY19L4*	1	1	1	1	0	1	1	6
miR-570-3p	*SCAI*	1	1	1	1	0	1	1	6
miR-570-3p	*EIF4E3*	0	1	1	1	1	1	1	6
miR-570-3p	*KRTAP11-1*	1	1	1	1	0	1	1	6
miR-570-3p	*LUZP2*	1	1	1	1	0	1	1	6
miR-570-3p	*ZDHHC21*	1	1	1	1	0	1	1	6
miR-570-3p	*KIAA2022*	1	1	1	1	0	1	1	6
miR-570-3p	*ZC3H12B*	1	1	1	1	0	1	1	6
miR-570-3p	*ZKSCAN2*	1	1	1	1	0	1	1	6
miR-570-3p	*OSTN*	1	1	1	1	0	1	1	6
miR-570-3p	*PLCXD3*	1	1	1	1	0	1	1	6
miR-570-3p	*MACC1*	1	1	1	1	0	1	1	6
miR-570-3p	*SKA2*	1	1	1	1	0	1	1	6
miR-570-3p	*C1orf95*	1	1	1	1	0	1	1	6

Table S1: Continued

miRNA	Gene	miRWalk	Microt4	miRanda	miRMap	RNA22	RNAhybrid	Targetscan	SUM
miR-570-3p	*PTAR1*	1	1	1	1	0	1	1	6
miR-570-3p	*ERCC6L2*	1	1	1	1	0	1	1	6
miR-570-3p	*CEP85L*	1	1	1	1	0	1	1	6
miR-570-3p	*FIBIN*	0	1	1	1	1	1	1	6
miR-570-3p	*TNFAIP8L3*	1	1	1	1	0	1	1	6
miR-570-3p	*PLEKHM3*	1	1	1	1	0	1	1	6
miR-570-3p	*VGLL3*	1	1	1	1	0	1	1	6
miR-570-3p	*LIN28B*	1	1	1	1	0	1	1	6
miR-570-3p	*IYD*	1	1	1	1	0	1	1	6
miR-570-3p	*NUDT19*	1	1	1	1	0	1	1	6
miR-570-3p	*C20orf202*	1	1	1	1	0	1	1	6
miR-570-3p	*CCSER1*	1	1	1	1	0	1	1	6
miR-570-3p	*RGS7BP*	1	1	1	1	0	1	1	6
miR-570-3p	*SAMD12*	1	1	1	1	0	1	1	6
miR-570-3p	*MINOS1*	1	1	1	1	0	1	1	6
miR-570-3p	*PALM2-AKAP2*	1	1	1	1	0	1	1	6
miR-570-3p	*CCDC73*	1	1	1	1	0	1	1	6
miR-570-3p	*CLLU1*	1	1	1	1	0	1	1	6
miR-570-3p	*ZNF704*	1	1	1	1	0	1	1	6
miR-570-3p	*C15orf56*	0	1	1	1	1	1	1	6
miR-570-3p	*SYNDIG1L*	1	1	1	1	0	1	1	6
miR-570-3p	*FAM198A*	0	1	1	1	1	1	1	6
miR-570-3p	*TMEM170B*	1	1	1	1	0	1	1	6
miR-570-3p	*C17orf96*	1	1	1	1	0	1	1	6
miR-570-3p	*TMEM178B*	1	1	1	1	0	1	1	6
miR-570-3p	*ALDH1A3*	1	0	1	1	1	1	1	6
miR-570-3p	*FOXN3*	1	0	1	1	1	1	1	6
miR-570-3p	*CLU*	1	0	1	1	1	1	1	6
miR-570-3p	*ETV1*	1	0	1	1	1	1	1	6
miR-570-3p	*FYN*	1	0	1	1	1	1	1	6
miR-570-3p	*GFPT1*	1	0	1	1	1	1	1	6
miR-570-3p	*GCLC*	1	0	1	1	1	1	1	6
miR-570-3p	*GNA12*	1	0	1	1	1	1	1	6
miR-570-3p	*ITPKB*	1	0	1	1	1	1	1	6
miR-570-3p	*DNAJB9*	1	0	1	1	1	1	1	6
miR-570-3p	*MYCL*	1	0	1	1	1	1	1	6
miR-570-3p	*NAP1L1*	1	0	1	1	1	1	1	6
miR-570-3p	*NCBP1*	1	0	1	1	1	1	1	6
miR-570-3p	*SERPINA1*	1	0	1	1	1	1	1	6
miR-570-3p	*PKP1*	1	0	1	1	1	1	1	6

Table S1: Continued

miRNA	Gene	miRWalk	Microt4	miRanda	miRMap	RNA22	RNAhybrid	Targetscan	SUM
miR-570-3p	*PMAIP1*	1	0	1	1	1	1	1	6
miR-570-3p	*PSMD9*	1	0	1	1	1	1	1	6
miR-570-3p	*SCN4B*	1	0	1	1	1	1	1	6
miR-570-3p	*SCN5A*	1	0	1	1	1	1	1	6
miR-570-3p	*SCN5A*	1	0	1	1	1	1	1	6
miR-570-3p	*SRL*	1	0	1	1	1	1	1	6
miR-570-3p	*CCL8*	1	0	1	1	1	1	1	6
miR-570-3p	*CNTN2*	1	0	1	1	1	1	1	6
miR-570-3p	*TGFB2*	1	0	1	1	1	1	1	6
miR-570-3p	*PRDM2*	1	0	1	1	1	1	1	6
miR-570-3p	*PRSS12*	1	0	1	1	1	1	1	6
miR-570-3p	*STX16*	1	0	1	1	1	1	1	6
miR-570-3p	*ADAM23*	1	0	1	1	1	1	1	6
miR-570-3p	*ANGPTL1*	1	0	1	1	1	1	1	6
miR-570-3p	*CDC42BPB*	1	0	1	1	1	1	1	6
miR-570-3p	*HS2ST1*	1	0	1	1	1	1	1	6
miR-570-3p	*SLC25A44*	1	0	1	1	1	1	1	6
miR-570-3p	*AREL1*	1	0	1	1	1	1	1	6
miR-570-3p	*ZBTB5*	1	0	1	1	1	1	1	6
miR-570-3p	*LPGAT1*	1	0	1	1	1	1	1	6
miR-570-3p	*STON1*	1	0	1	1	1	1	1	6
miR-570-3p	*CPSF6*	1	0	1	1	1	1	1	6
miR-570-3p	*PDZD2*	1	0	1	1	1	1	1	6
miR-570-3p	*TBC1D30*	1	0	1	1	1	1	1	6
miR-570-3p	*PPP1R13B*	1	0	1	1	1	1	1	6
miR-570-3p	*RASD2*	1	0	1	1	1	1	1	6
miR-570-3p	*NGFRAP1*	1	0	1	1	1	1	1	6
miR-570-3p	*RABGEF1*	1	0	1	1	1	1	1	6
miR-570-3p	*GOLT1B*	1	0	1	1	1	1	1	6
miR-570-3p	*TBC1D8B*	1	0	1	1	1	1	1	6
miR-570-3p	*SLC39A9*	1	0	1	1	1	1	1	6
miR-570-3p	*CNOT11*	1	0	1	1	1	1	1	6
miR-570-3p	*SMPD4*	1	0	1	1	1	1	1	6
miR-570-3p	*KANSL3*	1	0	1	1	1	1	1	6

Table S1: Continued

miRNA	Gene	miRWalk	Microt4	miRanda	miRMap	RNA22	RNAhybrid	Targetscan	SUM
miR-570-3p	*DNAJC11*	1	0	1	1	1	1	1	6
miR-570-3p	*IL36G*	1	0	1	1	1	1	1	6
miR-570-3p	*GPR108*	1	0	1	1	1	1	1	6
miR-570-3p	*SLC12A9*	1	0	1	1	1	1	1	6
miR-570-3p	*KIAA1210*	1	0	1	1	1	1	1	6
miR-570-3p	*NSD1*	1	0	1	1	1	1	1	6
miR-570-3p	*ZNF614*	1	0	1	1	1	1	1	6
miR-570-3p	*ORAI2*	1	0	1	1	1	1	1	6
miR-570-3p	*LDOC1L*	1	0	1	1	1	1	1	6
miR-570-3p	*C2orf88*	1	0	1	1	1	1	1	6
miR-570-3p	*SRRM4*	1	0	1	1	1	1	1	6
miR-570-3p	*FOXP2*	1	0	1	1	1	1	1	6
miR-570-3p	*PNMA5*	1	0	1	1	1	1	1	6
miR-570-3p	*ASB7*	1	0	1	1	1	1	1	6
miR-570-3p	*SLFN13*	1	0	1	1	1	1	1	6
miR-570-3p	*SPRED2*	1	0	1	1	1	1	1	6
miR-570-3p	*SDK1*	1	0	1	1	1	1	1	6
miR-570-3p	*KCTD20*	1	0	1	1	1	1	1	6
miR-570-3p	*CT45A3*	1	0	1	1	1	1	1	6
miR-570-3p	*CT45A4*	1	0	1	1	1	1	1	6
miR-570-3p	*CT45A2*	1	0	1	1	1	1	1	6
